# Structure-based, deep-learning models for protein-ligand binding affinity prediction

**DOI:** 10.1186/s13321-023-00795-9

**Published:** 2024-01-03

**Authors:** Debby D. Wang, Wenhui Wu, Ran Wang

**Affiliations:** 1 School of Science and Technology, Hong Kong Metropolitan University, 81 Chung Hau Sreet, Ho Man Tin, Hong Kong, China; 2 College of Electronics and Information Engineering, Shenzhen University, Shenzhen, 518060 China; 3 School of Mathematical Science, Shenzhen University, Shenzhen, 518060 China; 4 Guangdong Key Laboratory of Intelligent Information Processing, Shenzhen University, Shenzhen, 518060 China; 5 Shenzhen Key Laboratory of Advanced Machine Learning and Applications, Shenzhen University, Shenzhen , 518060 China

**Keywords:** Binding affinity prediction, Molecular representation, Deep learning, Interpretability, Structure-based drug discovery

## Abstract

The launch of AlphaFold series has brought deep-learning techniques into the molecular structural science. As another crucial problem, structure-based prediction of protein-ligand binding affinity urgently calls for advanced computational techniques. Is deep learning ready to decode this problem? Here we review mainstream structure-based, deep-learning approaches for this problem, focusing on molecular representations, learning architectures and model interpretability. A model taxonomy has been generated. To compensate for the lack of valid comparisons among those models, we realized and evaluated representatives from a uniform basis, with the advantages and shortcomings discussed. This review will potentially benefit structure-based drug discovery and related areas.

**Graphical Abstract:**

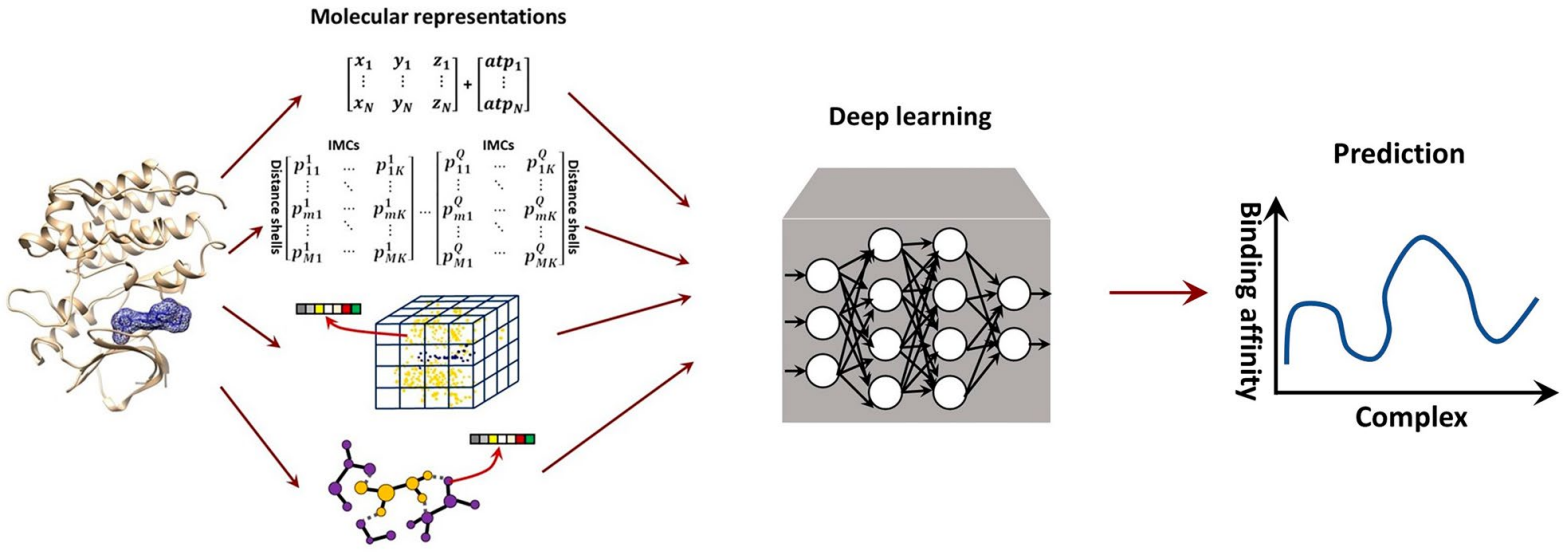

**Supplementary Information:**

The online version contains supplementary material available at 10.1186/s13321-023-00795-9.

## Introduction

Proteins, which frequently interact with other molecules to perform functions, are key participators in a wide spectrum of cellular processes. Interactions may occur between proteins and diverse ligand types, such as small organic molecules, nucleic acids and protein peptides. Particularly, inhibitors that bind to specific proteins to mediate disease progression (e.g. Gefitinib to EGFR protein in cancer therapies [1]) are examples of small-molecule ligands, making the interactions between such ligands and the target proteins a valuable objective of drug-development research.

Studies of protein-ligand interactions are mainly focused on the sites, modes or affinities of binding [2]. A drug-like ligand typically interacts with the target protein in a specific binding site (mostly a deep pocket), through a favorable binding orientation. The ligands that bind to the protein with high affinities are the initial aim of a drug-discovery pipeline. Determining the binding poses (site and orientation) for ligands to a target protein and estimating the binding affinities have therefore become two essential problems in computational drug discovery (CDD). Molecular docking is a well-developed class of computational methods that determine ligand-binding poses by efficiently searching the structural space and scoring the candidate poses [3]. Current docking methods can fastly produce binding poses that are quite close to the X-ray conformations (RMSD within 2$\mathring{A}$) [4], offering a possible alternative to experimentally resolved binding poses (e.g. by X-ray crystallography [5] and NMR spectroscopy [6]). A docking method commonly leverages a forcefield [7–11] to estimate the intermolecular forces (e.g. electrostatic interactions, van der Waals forces and desolvation effects), and recommends those binding poses with better forcefield scores. Although such scoring schemes are capable of measuring binding poses, they often fail in further tasks like distinguishing binders from non-binders and ranking the ligands for target proteins. Binding affinities, commonly quantified by dissociation constant ($K_d$) or inhibition constant ($K_i$), are more competent scores in these tasks. Effectively predicting such binding affinities is thus crucial, but has long been an open challenge in CDD.

Although a group of models for protein-ligand binding affinity prediction (PLBAP) rely on simple protein sequences and their evolutionary information (e.g. DeepDTA [12], DeepFusionDTA [13], GraphDTA [14] and CAPLA [15]), decoding the affinities from a deeper, structural perspective is always of high interests. The rapid release of protein-ligand binding structures (poses), by either docking engines or experimental techniques, provides a structural basis for rational PLBAP. Alongside the structural data, the increasingly revealed experimental affinity data (e.g. $K_{d/i}$ and *IC*50) [16, 17] has further facilitated supervised learning for PLBAP. Earlier machine-learning PLBAP models place a heavy emphasis on feature engineering, where protein-ligand interactions are estimated by domain-expertise-driven rules [18] or represented by exhaustive relevant factors [19, 20]. Later, there is a trend towards simplified feature engineering [21–24] and more powerful learning processes in PLBAP. Nevertheless, traditional machine-learning models (e.g. random forests and shallow neural networks) often have limited learning capabilities that hardly achieve favorable predictions.

In recent decades, deep neural networks (DNNs), which are credited with the strong learning capability on less engineered and unstructured data, have come into play in PLBAP. DNNs can absorb simple inputs, like atom coordinates and types [25] or statistics forms of pairwise atom-contacts [26], and learn them to predict protein-ligand binding affinity in an end-to-end manner. Beyond that, DNNs are prevalently used to learn geometric representations of protein-ligand complex structures [27, 28], such as voxelized grids [29] or molecular graphs [30], to provide high-quality PLBAP. Noteworthily, most of these works encounter heterogeneous data processing, coding platforms and validation procedures, calling for a comprehensive review and evaluation on them. On the other hand, although showing great potential in predictive accuracy in PLBAP, most DNNs are frequently questioned of their low interpretability. A reasonable discussion on their interpretabilities at the model level or in the post-hoc analysis stage [31–35] is the other goal of this work. Last but not least, there is a lack of exploring the screening performances of those deep-learning models in current works, bearing down on their practical value and requiring a study on their screening power. In what follows, we review mainstream deep-learning PLBAP models with a focus on the feature representations, learning architectures and interpretability. To compensate for the lack of valid and fair comparisons among them, a series of evaluations on the scoring and screening power of those models have been accomplished.

## Deep-learning PLBAP models

According to the feature representations and learning architectures, deep-learning PLBAP models are roughly categorized as in Table 1.

**Table 1:** Classification of deep-learning PLBAP models

Type	Feature representation ${\mathcal {R}}$	Symmetry properties$^*$ of ${\mathcal {R}}$	Key learning architecture	Model interpretability	Representatives
$T_{ACNN}$	Atom coordinates & types	TE/RE/PE	Concatenated ACNNs	Model-level	ACNN [25]
$T_{IMC-CNN}$	IMC profiles	TI/RI/PI	2D-CNNs	None	OnionNet [26], OnionNet-2 [36], IMCP-Score [37]
$T_{Grid-CNN}$	Grid voxels	TI/RE/PI	3D-CNNs	Post-hoc analysis	KDEEP [29], Pafnucy [38], CNN-Score [39], DeepAtom [40], Sfcnn [41]
$T_{Graph-GCN}$	Molecular graphs	TI/RI/PI	GCNs	Model-level	GraphBAR [30], APMNet [42], PotentialNet [43], GraphDTI [44]

$^{*}$ *TI* translation invariance, *RI* rotation invariance, *PI* atom permutation invariance

*TE* translation equivariance, *RE* rotation equivariance, *PE* atom permutation equivariance

### PLBAP based on $T_{ACNN}$ models

**Fig. 1:**
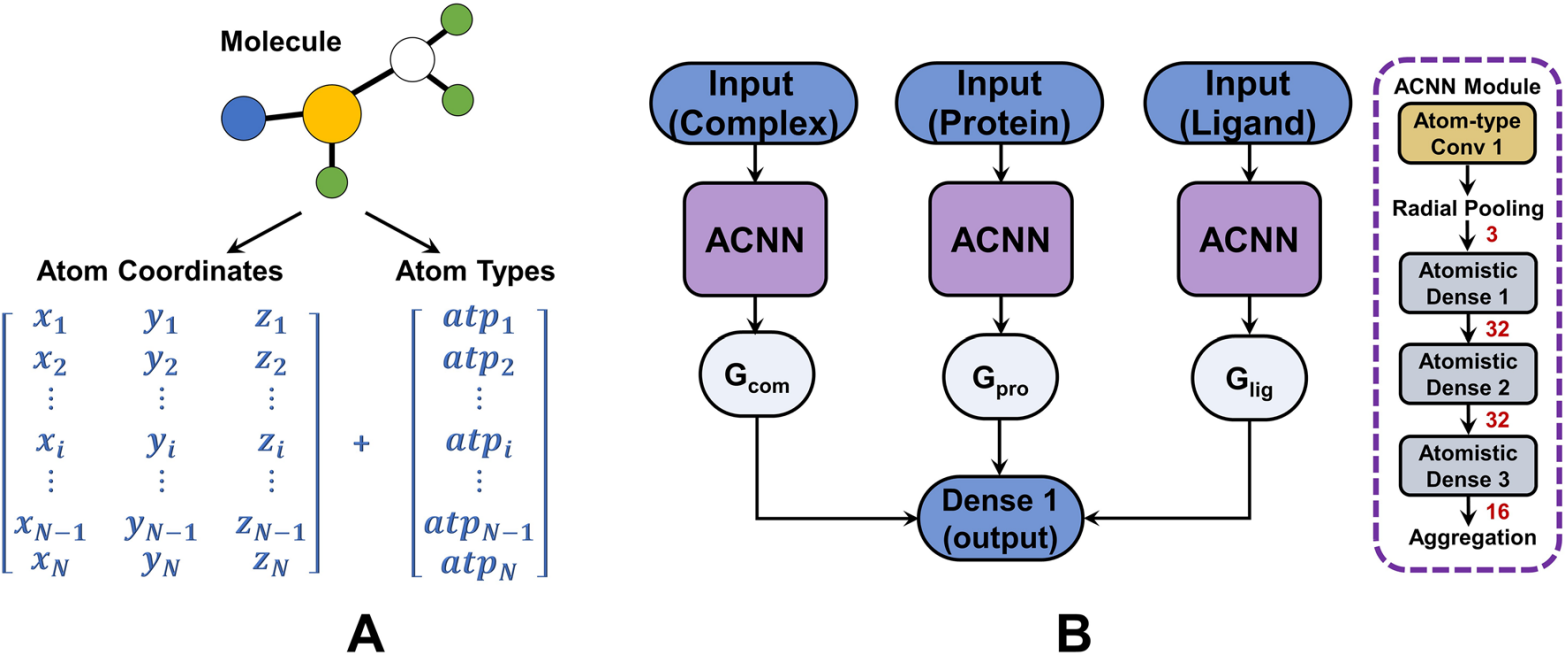
The inputs and learning architecture for ACNN-based PLBAP. **A** The inputs for an ACNN module. **B** The learning architecture for PLBAP. The red numbers indicate the number of filters in radial-pooling layer or the numbers of units in atomistic dense layers

Gomes and co-workers have devised Atomic Convolutional Neural Networks (ACNNs), which absorb the coordinates $\varvec{{\mathcal {C}}}=\{\varvec{{\mathcal {C}}}_i|i=1,\ldots ,N\}=\{(x_i, y_i, z_i)|i=1,\ldots ,N\}$ and types $\varvec{\mathcal {ATP}}=\{atp_i|i=1,\ldots ,N\}$ of atoms in a molecular structure (Fig. 1A) and output the estimated energy *E* of this molecule [25]. A molecule is represented by a feature tensor $\textbf{T}(i,j,k)$ outlining the local chemical environments of each atom. $\textbf{T}(i,j,k)$ is generated by applying atom-type convolutions to the distance matrix ($\in {\mathbb {R}}^{N\times M}$) [45] and atom-type matrix ($\in {\mathbb {R}}^{N\times M}$), which are derivatives of $\varvec{{\mathcal {C}}}$ and $\varvec{\mathcal {ATP}}$. It can be expressed as:

1 $$\begin{aligned} \textbf{T}(i,j,k) = {\left\{ \begin{array}{ll} \parallel {\mathcal {C}}_i-{\mathcal {C}}_{i_j}\parallel &{} atp_{i_j}=\omega _k\\ 0 &{} otherwise \end{array}\right. } \end{aligned}$$

where ${\mathcal {C}}_i$ represents the coordinates of the *i*-th atom $\textbf{a}_i$ ($i=1,\ldots ,N$), $\textbf{a}_{i_j}$ ($j=1,\ldots ,M$) is the *j*-th nearest spatial neighbor of $\textbf{a}_i$, and $\omega _k\in \Omega$ ($k=1,\ldots ,K$) indicates a specific atom type (e.g. C, O and N). Such a feature tensor ($\in {\mathbb {R}}^{N\times M\times K}$) is fed into a radial-pooling layer to prevent overfitting and reduce parameters. A pooling filter $f_q$ ($q=1,\ldots ,Q$) combines the pairwise interactions between an atom $\textbf{a}_i$ and its neighbors having a specific type $\omega _k$ as:

2 $$\begin{aligned} \begin{aligned} \textbf{P}(i,k,q) =&\sum \limits _{j=1}^M f_q(\textbf{T}(i,j,k))\\ where\ \ f_q(x) =&{\left\{ \begin{array}{ll} \frac{1}{2}e^{-\frac{(x-r_q)^2}{\sigma _q^2}}(cos(\frac{\pi x}{R_c})+1) &{} 0<x<R_c\\ 0 &{} x\ge R_c\\ \end{array}\right. } \end{aligned} \end{aligned}$$

where $R_c$ is a distance threshold (e.g. $12\mathring{A}$), and $r_q$ and $\sigma _q$ are learnable parameters. The feature tensor after pooling ($\in {\mathbb {R}}^{N\times K\times Q}$) is flattened and fed row-wise into several atomistic dense layers. Outputs for each row indicate the estimated atomic energy ($E_i$), and combining them yields the total estimated energy (*E*) of the molecule.

ACNN-based PLBAP adopts a learning architecture that implies a ligand-binding thermodynamic cycle (Fig. 1B). The binding affinity in this architecture is estimated as the energy difference between the complex and the two binding molecules ($y=\Delta G = G_{complex}-G_{protein}-G_{ligand}$). As reported in this work, simply employing 15 atom types (C, N, O, F, Na, Mg, P, S, Cl, Ca, Mn, Zn, Br, I and others regarded as a single type), 3 radial filters ($r_q$ = 0, 4.0 or 8.0, $\sigma _q^2$ = 2.5) and 3 atomistic dense layers (sizes of 32, 32 and 16) can yield state-of-the-art prediction performances (validated on *PDBbind* benchmarks).

*Model Interpretability *$T_{ACNN}$ models possess a hierarchical structural of model-level interpretability. The atom-type convolutions and radial pooling operations lead to the estimation of atomic pairwise interactions, providing the interpretability at an elementary level. The atomistic fully connected layers then increase this interpretability to a molecular level, by accumulating pairwise interaction energies into the total energy of a molecule. At the top level, a thermodynamic cycle of ligand-binding process is imposed to achieve an overall interpretability in physico-chemical mechanisms.

### PLBAP based on $T_{IMC-CNN}$ models

This category represents protein-ligand interactions with intermolecular contacts (IMCs), and feeds the re-organized features (e.g. matrices) to 2-dimensional convolutional neural networks (2D-CNNs) for learning the data relationships. An intermolecular contact is defined as a pair of atoms, one from the protein ${\textbf{a}}_i^P$ and the other from the ligand ${\textbf{a}}_j^L$, within a distance threshold $d_{cut}$ [21]. Considering all atom types for the protein ($\Omega ^P$) and ligand ($\Omega ^L$), it leads to $M=|\Omega ^P|\times |\Omega ^L|$ types of IMCs. These IMCs can be further refined using the concept of shell space [26]. Regarding $\textbf{a}_j^L$ as a spherical center, the space between two spherical boundaries (with radii of $d_{cut1}$ and $d_{cut2}$) forms a shell and any protein atom $\textbf{a}_i^P$ within this shell will form a refined IMC with $\textbf{a}_j^L$. For a protein-ligand complex, *M* IMC types $\Omega ^{IMC}=\{\omega ^{IMC}_m\}=\{(\omega _1^m,\omega _2^m)|\omega _1^m \in \Omega ^P,\omega _2^m\in \Omega ^L,m=1,\ldots ,M\}$ and *K* distance shells $\Delta =\{\delta _k\}=\{(d_{cut1}^k,d_{cut2}^k]|k=1,\ldots ,K\}$ result in a feature matrix ($\in {\mathbb {R}}^{M\times K}$) exhibiting multi-range intermolecular interactions (Eq. 3).

3 $$\begin{aligned} \begin{aligned} \textbf{F}_{OnionNet}(m, k)&= \sum \limits _{i,j} I_{m, k}({\textbf{a}}_i^P, {\textbf{a}}_j^L)&\\ with\ \ I_{m, k}({\textbf{a}}_i^P, {\textbf{a}}_j^L) =&{\left\{ \begin{array}{ll} 1 &{} (atp_i^P, atp_j^L) = \omega ^{IMC}_m\ \& \ \parallel {\mathcal {C}}_i^P-{\mathcal {C}}_j^L\parallel \in \delta _k\\ 0 &{} otherwise \end{array}\right. } \end{aligned} \end{aligned}$$

OnionNet employs $K=60$ shells spanning from 0 to $30\mathring{A}$ ($\delta _1=(0,1\mathring{A}]$, $\delta _2\sim \delta _{60}$ with fixed intervals of $0.5\mathring{A}$), and 8 types for both protein and ligand atoms ($\Omega ^P=\Omega ^L=\{$C, N, O, H, P, S, HAX and Du$\}$) to identify IMCs. Similarly, OnionNet-2 profiles the contacts between protein residues and ligand atoms in different distance shells [36]. Regarding each type of IMCs ($\omega ^{IMC}_m$) within a distance shell ($\delta _k$) as a specific type of interactions, we can profile these interactions using quantities, average contact distances and other properties (e.g. pharmacophoric features). IMCP-Score [37] simply profiles such interactions by quantity of the contacts and the average atomic distances of them (Eq. 4).

4 $$\begin{aligned} \textbf{F}_{IMCP}(m, k) = (p_1,p_2)=(\sum \limits _{i,j} I_{m, k}({\textbf{a}}_i^P, {\textbf{a}}_j^L),\frac{\sum \limits _{i,j} I_{m, k}({\textbf{a}}_i^P, {\textbf{a}}_j^L)~\cdot \parallel {\mathcal {C}}_i^P-{\mathcal {C}}_j^L\parallel }{\sum \limits _{i,j} I_{m, k}({\textbf{a}}_i^P, {\textbf{a}}_j^L)}) \end{aligned}$$

**Fig. 2:**
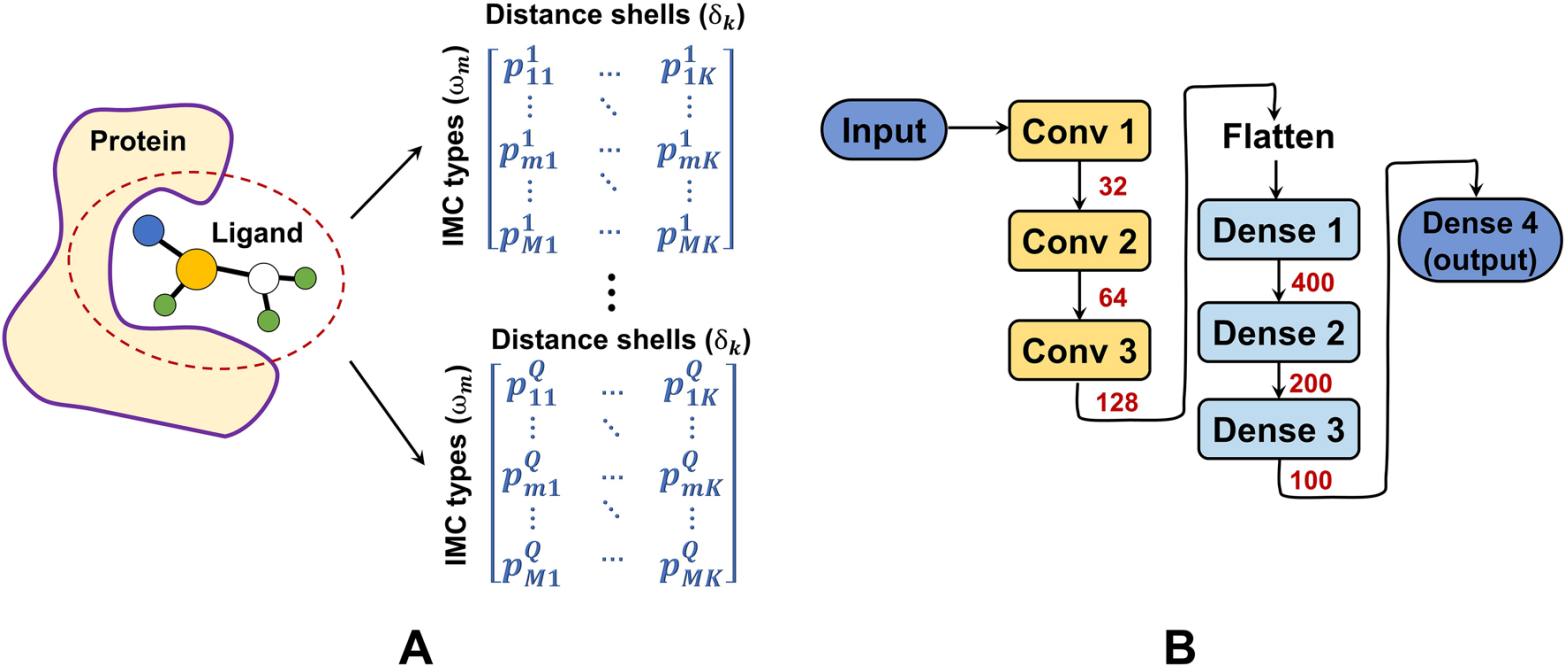
The feature representation and learning architecture of $T_{IMC-CNN}$ models. **A** The feature representation. **B** The learning architecture used by OnionNet for PLBAP. The red numbers indicate the numbers of filters in convolution layers or the numbers of units in dense layers

IMC-based features can be arranged as matrices or tensors (Fig. 2A) to be fed into 2D-CNNs. Conventional 2D-CNN architectures are commonly adopted for learning these features, and Fig. 2B presents the one used by **OnionNet** [26]. It includes 3 consecutive convolution layers ($4\times 4$ kernels with stride 1), 1 flattening layer, 3 consecutive dense layers (400, 200 and 100 units) and 1 output layer. In the model-training phase, a customized loss function, involving both the Person’s correlation coefficient and the root-mean-square error, is adopted by OnionNet. This category of models are easy to generate, and have led to competitive PLBAP (validated on *PDBbind* benchmarks).

Model Interpretability: Although neither model-level nor post-hoc interpretability was provided in the original works of $T_{IMC-CNN}$ Models, they can be partly explained in a post-hoc manner, such as by measuring the feature importance in affinity predictions.

### PLBAP based on $T_{Grid-CNN}$ models

This category leverages molecular grids to represent protein-ligand complexes, and employs three-dimensional CNNs (3D-CNNs) to learn the grids. The molecular grid representation of a protein-ligand complex structure $\varvec{{\mathcal {S}}}$ emphasizes the binding area instead of the whole structure, in order to ease the computational burden. It captures the features of the binding area at regularly spaced intervals (resolution). Suppose the binding area of $\varvec{{\mathcal {S}}}$ is represented as a grid with the size of $X\mathring{A}\times Y\mathring{A}\times Z\mathring{A}$ and the resolution of $r\mathring{A}$. Each cell $\textbf{c}$ ($r\mathring{A}\times r\mathring{A}\times r\mathring{A}$) in the grid is delineated as a feature vector $\textbf{f}^\textbf{c} = (f_1^\textbf{c}, f_2^\textbf{c}, \ldots , f_K^\textbf{c})$, indicating a multi-channel voxel. Integrating all these voxels leads to a 4D tensor as follows,

5 $$\begin{aligned} \textbf{F}(x,y,z) = \textbf{f}^{\textbf{c}}\ with\ {\left\{ \begin{array}{ll} -\frac{X}{2}< x< \frac{X}{2} \\ -\frac{Y}{2}< y< \frac{Y}{2} \\ -\frac{Z}{2}< z < \frac{Z}{2} \end{array}\right. } \end{aligned}$$

Here (*x*, *y*, *z*) indicates the center of $\textbf{c}$. Given a complex structure and the grid size (e.g. $X=Y=Z=24$ and $r=1$ in KDEEP [29]), the key to constructing a molecular grid is properly assigning features to each cell.

All $T_{Grid-CNN}$ models start from the atom-level features. They mostly cover general properties (e.g. atom types) [29, 38, 39, 41, 46], physico-chemical properties (e.g. excluded volume, partial charge, heavy-atom neighbors, hetero-atom neighbors, and hybridization) [29, 38, 46] and pharmacophoric properties (e.g. hydrophobicity, aromaticity, H-bond donor/acceptor, and ring member) [29, 38–40, 46]. These properties are commonly estimated by SMARTS patterns [47, 48] or simple geometric rules [48, 49]. Each atom $\textbf{a}_i$ is characterized by *K* properties as $\textbf{p}^{\textbf{a}_i} = (p_1^{\textbf{a}_i}, p_2^{\textbf{a}_i}, \ldots , p_K^{\textbf{a}_i})$, which can be used to fill in the molecular grid having a coincident center with the ligand. There are two common strategies for filling information in the grids. KDEEP, DeepAtom and CNN-Score adopt an expensive method that measures the contributions of each atom $\textbf{a}_i$ to each cell $\textbf{c}_j$ and accumulates the contributions for $\textbf{c}_j$. As an instance, KDEEP quantifies the contributions by Euclidean distances and calculates the *k*-th channel feature of cell $\textbf{c}_j$ as Eq. 6.

6 $$\begin{aligned} f^{\textbf{c}_j}_k = \sum \limits _{i} (1-e^{-(\frac{r_{VDW}^{\textbf{a}_i}}{\Vert {\mathcal {C}}_i^A-{\mathcal {C}}_j^C\Vert })^{12}})p_k^{\textbf{a}_i} \end{aligned}$$

**Fig. 3:**
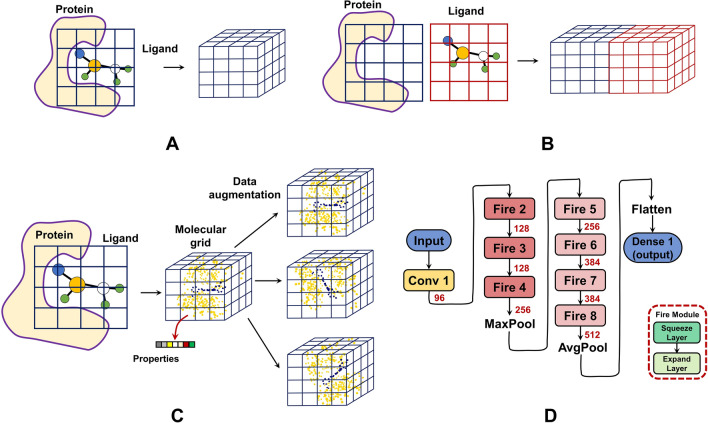
The grid representation and learning architecture of $T_{Grid-CNN}$ models. **A** One grid that covers both protein and ligand atoms. **B** Two concatenated grids that featurize protein and ligand atoms separately. **C** An augmented grid representation. **D** The learning architecture for PLBAP (used by KDEEP). The red numbers indicate the numbers of filters in convolution layers or the numbers of units in dense layers

Where $r_{VDW}^{\textbf{a}_i}$ is the van der Waals radius of $\textbf{a}_i$, and ${\mathcal {C}}_i^A$ and ${\mathcal {C}}_j^C$ are coordinates of the centers of $\textbf{a}_i$ and $\textbf{c}_j$. Another strategy is simply aggregating the features for atoms located in each cell. Pafnucy, DeepFusionNet [46] and Sfcnn employ this strategy, which is efficient but may lead to low interpretability (e.g. for categorical features). Given a grid-filling strategy, a complex can be represented by one filled grid covering all protein and ligand atoms (Fig. 3A), or two concatenated grids treating protein and ligand atoms separately (Fig. 3B). Due to the lack of rotation invariance of grid representations, data augmentation by rotating the grids is frequently adopted to strengthen the data (Fig. 3C).

The learning architectures employed by this category include simple (similar to Fig. 2B) [38], self-developed [41] or well-developed architectures in other fields (e.g. *SqueezeNet* [29, 50], *ShuffleNet* [40, 51] and *Caffe* [39, 52]). As demonstrated in the work of **Sfcnn**, going deeper in CNN architectures did not promote prediction improvements. Considering the large resources (augmented grids) consumed here, light-weight learning architectures like SqueezeNet (used by KDEEP) is a fine option. SqueezeNet was first developed to compress the learnable parameters in earlier architectures like AlexNet [53], and inspired the architecture of KDEEP exceedingly (Fig. 3D). The grid representations will first go through a convolution layer ($7\times 7\times 7$ kernels with stride 2) and a series of fire modules before the final output layer. Each fire module is composed of a squeeze layer (*n* $1\times 1\times 1$ kernels) and an expand layer (4*n* $1\times 1\times 1$ and 4*n* $3\times 3\times 3$ kernels). For instance, Fire2 module involves 16 kernels in squeeze layer and 128 kernels (64 $1\times 1\times 1$ and 64 $3\times 3\times 3$ kernels) in expand layer. The pooling layers combine $3\times 3\times 3$ voxels at strides of 2. This category plays a major role in deep-learning PLBAP models (validated on *PDBbind* benchmarks), while may be limited to the expensive computations.

Model Interpretability: KDEEP and DeepAtom lack both model-level and post-hoc interpretability [29, 40]. CNN-Score provides a visualization strategy for evaluating prediction-level post-hoc interpretability. It applies masking [54] to various regions in a grid, and the masking-induced differences in predicted scores yield a heat-map revealing important regions. Crucial residues in the binding area are often highlighted in such analyses, implying that CNN-Score predicts binding affinities based on key features of protein-ligand interactions. Pafnucy adopts two ways in post-hoc interpretability analysis. L2-regularized model-training provides the profile of feature importance by showing the weight distributions of the first-hidden-layer convolutional filters. Wider-range weights are proposed to pass more information to the deeper layers and therefore have greater impact on the predictions. Aside from above dataset-level interpretations, Pafnucy also provides a voxel-removal strategy for prediction-level interpretations. By removing voxels ($5\mathring{A}\times 5\mathring{A}\times 5\mathring{A}$) at different positions in the featurization area ($20\mathring{A}\times 20\mathring{A}\times 20\mathring{A}$), the resulted prediction changes were investigated further. Key intermolecular interactions (e.g. Hydrogen bond, $\pi$-$\pi$ interaction and hydrophobic contacts) were revealed by such analysis. Sfcnn was explained at the prediction-level, by hot-spot areas of the input features that are closely related to the predictions [41]. These hot-spot areas or heat-maps were generated based on gradient-weighted class activation mapping (Grad-CAM) analysis [55] and visualized using Mayavi [56]. As uncovered in the work of Sfcnn, such hot-spot areas highly corresponded to important protein-ligand interactions like hydrophobic contacts and hydrogen bonds.

### PLBAP based on $T_{Graph-GCN}$ models

This group of models represent a protein-ligand complex by a graph $\{\textbf{V}, \textbf{E}\}$, where $\textbf{V}$ indicates the nodes and $\textbf{E}$ the edges. (i) For PLBAP, $\textbf{V}=\{\textbf{a}_i|i=1,\ldots ,N\}$ generally covers all the ligand atoms and the atoms in the ligand-binding site of the protein (e.g. those within a predefined distance from any ligand atom). Practically, a fixed number *N* for a set of complexes, such as $N=200$ adopted by GraphBAR [30], is required for batch-computations. Each $\textbf{a}_i\in \textbf{V}$ is characterized by *M* atom-level features that resemble those in grid representations (Sect. [Sec Sec4]), leading to a node-feature matrix ${\mathcal {M}}_V\in {\mathbb {R}}^{N\times M}$ for each complex. (ii) Originally, $\textbf{E}$ of a molecular graph encompasses all the covalent bonds, which can be encoded in an adjacency matrix $\textbf{A}\in {\mathbb {R}}^{N\times N}$ with $\textbf{A}_{ij}=1$ signifying a chemical bond between atoms $\textbf{a}_i$ and $\textbf{a}_j$. As an instance, APMNet [42] considers the covalent bonds as $\textbf{E}$ in the graph representations for PLBAP. However, the binding between a protein and its ligands counts heavily on noncovalent interactions, such as hydrogen bonds and $\pi -\pi$ stacking. It necessitates the generalization of $\textbf{A}$ to an adjacency tensor (${\mathbb {R}}^{N\times N\times N_{et}}$) as below.

7 $$\begin{aligned} \textbf{A}_{ijk}={\left\{ \begin{array}{ll} 1 &{} \textbf{a}_i~and~ \textbf{a}_j~have~an~ edge~of~type~k\\ 0 &{} otherwise \end{array}\right. } \end{aligned}$$

Where $N_{et}$ is the number of edge types, and any slice of the tensor $\textbf{A}_{::k}$ indicates a specific type of adjacency. Different from the chemical bonds, noncovalent interactions are commonly determined according to pairwise atomic distances below some threshold values. **PotentialNet** [43] uses the first slice $\textbf{A}_{::1}$ to show covalent adjacency, while the following $\textbf{A}_{::k}$ $(k\ge 2)$ to indicate noncovalent interactions identified by distance thresholds (e.g. $<3\mathring{A}$). **GraphBAR** [30] relies on $N_{et}$ distance shells $\Delta =\{\delta _k\}=\{(\frac{4(k-1)}{N_{et}}, \frac{4k}{N_{et}}]|k=1,\ldots ,N_{et}\}$, and assigns $\textbf{A}_{ijk}=1$ if the distance between $\textbf{a}_i$ and $\textbf{a}_j$ falls in the *k*-th shell. **DeepFusionNet** [46] adopts two distance shells $\Delta =\{\delta _1,\delta _2\}=\{(0, 1.5], (1.5, 4.5]\}$ to discriminate between covalent and noncovalent adjacencies, and directly utilizes the atomic distances as the adjacency values (Eq. 8).

8 $$\begin{aligned} \textbf{A}_{ijk}={\left\{ \begin{array}{ll} \Vert {\mathcal {C}}_i-{\mathcal {C}}_j\Vert &{} \Vert {\mathcal {C}}_i-{\mathcal {C}}_j\Vert \in \delta _k\\ 0 &{} otherwise \end{array}\right. } \end{aligned}$$

Similarly, **GraphDTI** [44] presents the covalent adjacency by the first slice $\textbf{A}_{::1}$ (logical), while combines the covalent and noncovalent interactions within $5\mathring{A}$ in $\textbf{A}_{::2}$ (Eq. 9).

9 $$\begin{aligned} \textbf{A}_{ij2}={\left\{ \begin{array}{ll} 1 &{} \textbf{a}_i~and~\textbf{a}_j~ are~covalently~bonded\\ e^{-\frac{(\Vert {\mathcal {C}}_i-{\mathcal {C}}_j\Vert -\mu )^2}{\sigma }} &{} \Vert {\mathcal {C}}_i-{\mathcal {C}}_j\Vert \le 5~ \& ~no~covalent~bond~between~\textbf{a}_i~and~\textbf{a}_j\\ 0 &{} otherwise \end{array}\right. } \end{aligned}$$

**Fig. 4:**
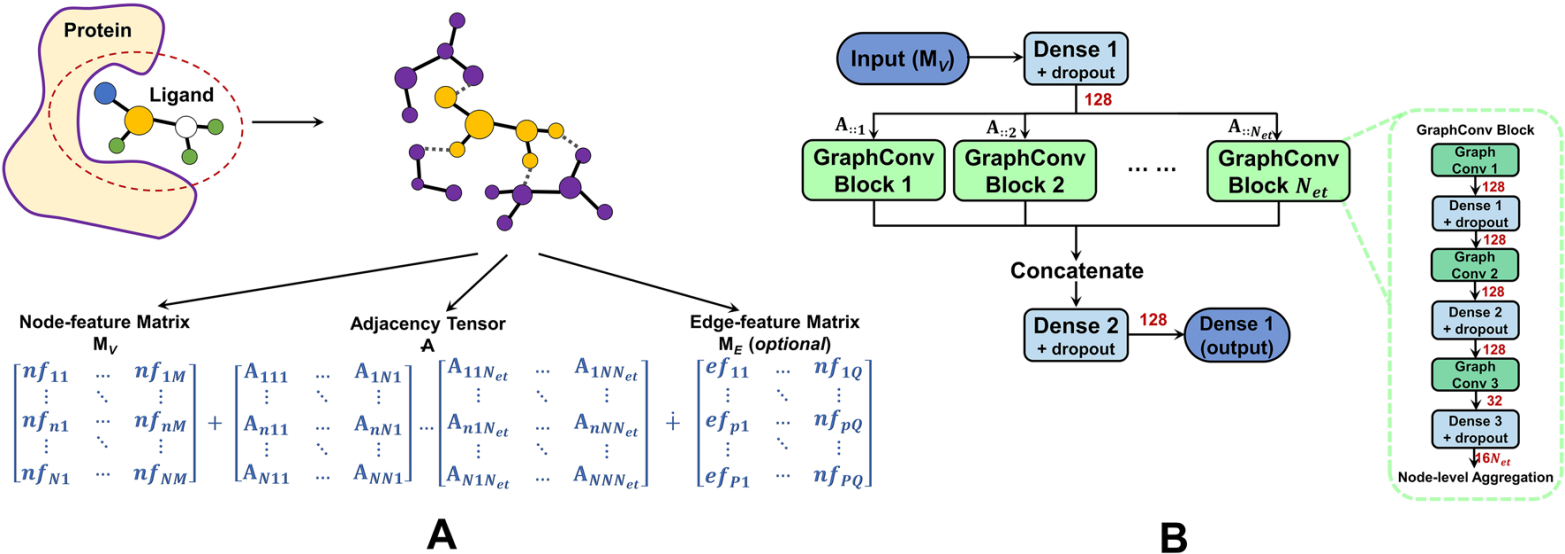
The graph representation and learning architecture of $T_{Graph-GCN}$ models. **A** A schematic diagram of graph representation. **B** The learning architecture for PLBAP (used by GraphBAR). The red numbers indicate the numbers of filters in graph convolution layers or the numbers of units in dense layers

Here the adjacency values for noncovalent interactions are weaker than those for covalent bonds. Beyond above, some models (e.g. APMNet [42]) further characterize the edges by one-hot encoding of multiple bond types (e.g. single, double and triple bonds), leading to an edge-feature matrix ${\mathcal {M}}_E$. A schematic diagram of graph representations is displayed in Fig. 4A. Models like PLANET [57] and GraphscoreDTA [58] treat protein residues as nodes and connect consecutive residues by edges, which result in simple 1D graphs and are regarded as sequence-based. Accordingly, they are out of scope for this review.

Molecular graph representations that are invariant to rotations [27, 28] can be learned by Graph Convolutional Networks (GCNs) [59–61]. Most GCNs adopt a message-passing mechanism, which iteratively updates the features of each node ($h_i^{t+1}$) by gathering information from its neighborhood ($r_i^{t+1}$) and generates a graph-level feature vector ($\hat{f}$) based on updated node features. This process can be expressed as follows.

10 $$\begin{aligned} {\left\{ \begin{array}{ll} r_i^{t+1} &{} = \sum \limits _{\textbf{a}_j\in Nr(\textbf{a}_i)}MP_t(h_i^t,h_j^t) \\ h_i^{t+1} &{} = U_t(h_i^t,r_i^{t+1}) \\ \hat{f} &{} = Gr(\{h_i^T|\textbf{a}_i\in \textbf{V}\}) \end{array}\right. } \end{aligned}$$

where $h_i^0$ comes from the initial node features ${\mathcal {M}}_V$, $Nr(\textbf{a}_i)$ indicates all the neighboring atoms of $\textbf{a}_i$ upon a specific type of adjacency, *T* is the number of iterations, and $MP_t$, $U_t$ and Gr (permutation-invariant) are learned functions that differentiate among various GCN models. GraphBAR relies on a spectral GCN architecture (Fig. 4B) to learn the molecular graphs. The node-feature matrix ${\varvec{{\mathcal {M}}}}_V$ is preprocessed (by dense layer with 128 units and a dropout rate of 0.5) before going into graph convolutional blocks $GCB_k$ ($k=1,\ldots ,N_{et}$). The fundamental propagation rule for layers in $GCB_k$ is $\textbf{H}_k^{t+1} =\sigma (\textbf{L}_k\textbf{H}^{t}_k\Theta ^t_k)$, where $\textbf{H}^{t}_k$ is the node-feature matrix of the *t*-th layer, $\Theta _k$ is a matrix of trainable parameters ($\in {\mathbb {R}}^{N_{in}\times N_{out}}$), $\sigma (\cdot )$ indicates an activation function (e.g. *ReLU*) and $\textbf{L}_k$ cencerns the *k*-th type of adjacency ($\textbf{L}_k=\textbf{D}^{-\frac{1}{2}}\tilde{\textbf{A}}^k\textbf{D}^{-\frac{1}{2}}=\textbf{D}^{-\frac{1}{2}}(\textbf{A}_{::k}+\textbf{I}_N)\textbf{D}^{-\frac{1}{2}}$ with $\textbf{D}_{ii}=\sum _{j}\tilde{\textbf{A}}^k_{ij}$). Each $GCB_k$ includes three convolutional layers (128, 128 and 32 filters) and three dense layers (128, 128 and $16N_{et}$ units) with a dropout rate of 0.5. Aggregating all node features in $GCB_k$ ($\hat{f}_k$), concatenating them ($\mathbin \Vert _{k}\hat{f}_k$) and connecting them to a dense layer (128 units with dropout) finally lead to the output of binding affinity. APMNet primarily involves two message-passing modules in its learning architecture. Module 1 includes a series of graph convolutional skip blocks $GCSB_k$, with each block considering the intial node-feature matrix (${\mathcal {M}}_V$) and sharing the weights during feature propagations. The outputs $\textbf{H}^{T}_k$ from $GCSB_k$ ($k=1,\ldots ,K$) in module 1 are averaged ($\bar{\textbf{H}}$) and fed into module 2 for further learning, with ${\mathcal {M}}_E$ taken into consideration. The outputs of module 2 are aggregated at the node-level and connected to the dense/output layer for PLBAP. PotentialNet connects two gated graph neural network (GGNN) modules in a cascade way, and gathers the graph features at a node-level (ligand atoms only) to feed them into a number of dense layers. GraphDTI [44] leverages the gated graph attention (distance-aware) layers to update node features and learn noncovalent interactions at the binding site. The updated features after T layers for all ligand atoms are aggregated and fed to dense layers for predictions. Favorable PLBAP performances have been yielded from this category of models (validated on PDBbind benchmarks).

Model Interpretability: GraphBAR is to some extent explainable at the model-level. Each filter corresponding to $\textbf{A}_{::k}$ convolves the first-order neighborhood of a node and generates related node features. Summed features of all nodes (row-wise aggregation of $\textbf{H}_k^T$) imply specific protein-ligand interactions in the binding site, and concatenating various interactions for a protein-ligand pair then leads to the total binding affinity. Analogously, other models such as APMNet and GraphDTI can also be interpreted at the model-level from the perspective of energies. Beyond that, these models can also be explained by measuring the feature importance in the predictions, as a post-hoc analysis.

## Evalution of models

### Evaluation of scoring performances

To generally evaluate the four types of models ($T_{ACNN}$, $T_{IMC-CNN}$, $T_{Grid-CNN}$ and $T_{Graph-GCN}$), we have constructed representatives using the uniform training data and property-generation rules.*** Training and validation data.*** The frequently accessed *PDBbind Refined Set* (V2020) [16, 62] was employed for model training, with the *Core Set* used for hyperparameter tuning. Two CSAR-HiQ data sets [63, 64] from another source were adopted for testing the models. These sets (details in Additional file 1: Table S1) are all comprised of experimentally determined protein-ligand complex structures with their binding constants ($K_{d/i}$). The original sizes of them are 5,316 for *Refined Set*, 285 for *Core Set*, 175 for *CSAR-HiQ Set 1* and 167 for *CSAR-HiQ Set 2*, respectively. 460 overlapped complexes between the *Refined Set* and the others were removed from the *Refined Set*, resulting in a final training set of 4856 complexes. A PLBAP model attempts to correlate the structure of a protein-ligand complex with the binding affinity ($-\log K_{d/i}$ in this study). ***Atomic property generation.*** General and pharmacophoric properties of atoms in the protein-ligand complexes were generated by OpenBabel [65] and RDKit [66]. Standing on atomic properties, different molecular representations for $T_{ACNN}$, $T_{IMC-CNN}$, $T_{Grid-CNN}$ and $T_{Graph-GCN}$ models can be generated. ***Model training.*** Given a feature representation (e.g. atom coordinates/types, IMC matrix, grid or graph), we mainly tuned the parameters (e.g. batch size bs and number of epochs epc) related to the training process, with the majority of model parameters fixed (from well-validated architectures). The learning architectures were realized using Tensorflow with the loss function of mean squared error and the optimizer of Adam. Hyperparameters were tuned by KerasTuner, and all computations were GPU-accelerated. Model construction details can be found in the Additional file. ***Evaluation rules.*** Pearson’s Correlation (PC) and root-mean-squared error (RMSE) between the predicted and true binding affinities were adopted as the evaluation indices. A higher PC and a lower RMSE indicate a better prediction performance.

**Fig. 5:**
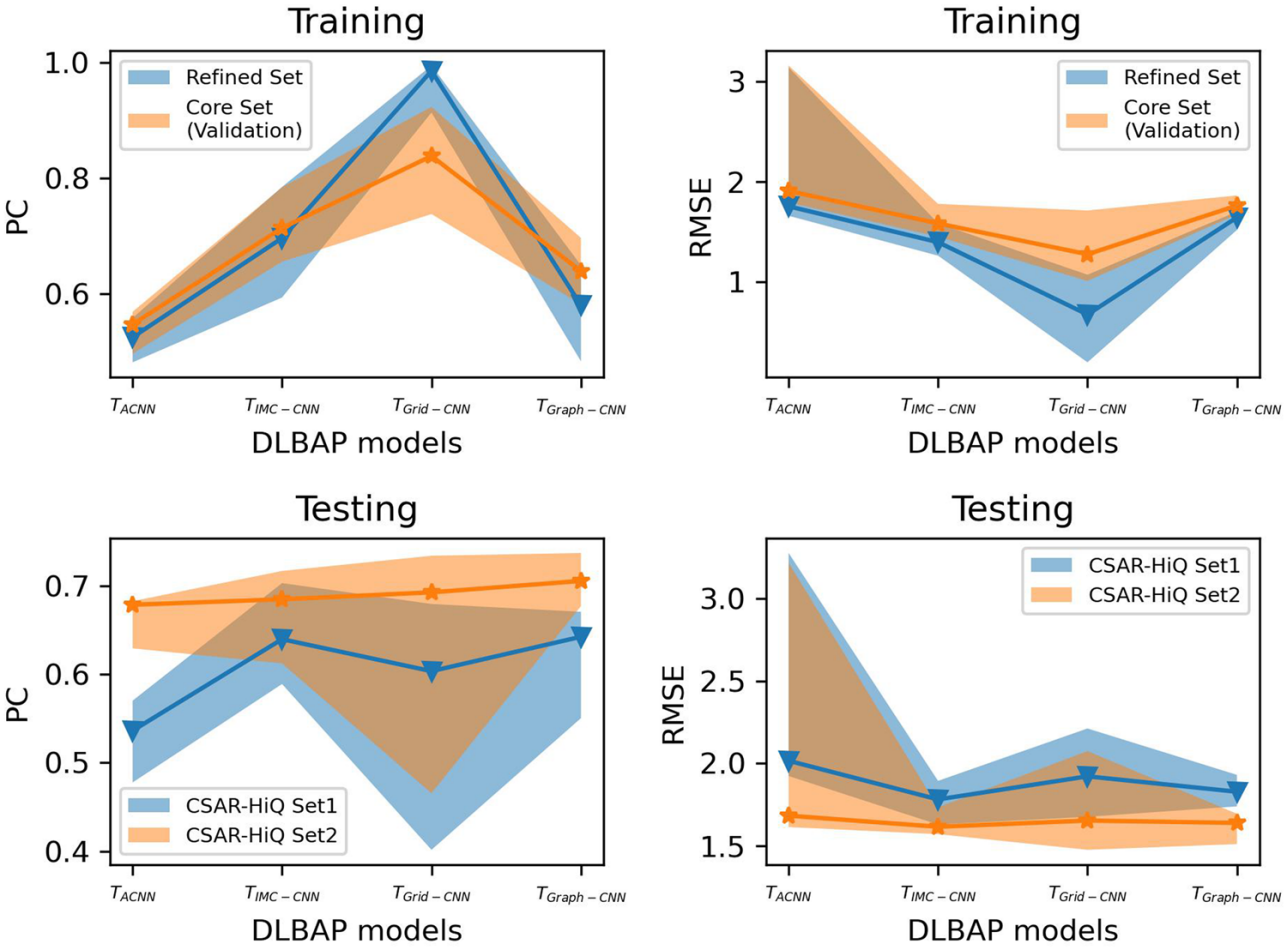
Scoring performances of representative deep-learning PLBAP models. The models were trained on *PDBbind Refined Set*, validated on the *Core Set* (for hyperparameter tuning) and finally tested on two CSAR-HiQ sets. The lines show the median values of each type of models

By combining different feature representations with various model architectures, we have trained 26 representatives ($M_1\sim M_{26}$) belonging to the four types of models ($T_{ACNN}$: $M_1\sim M_6$, $T_{IMC-CNN}$: $M_7\sim M_{10}$, $T_{Grid-CNN}$: $M_{11}\sim M_{18}$ and $T_{Graph-GCN}$: $M_{19}\sim M_{26}$). The scoring performances of these models (details in Additional file 2: Table S2) are now presented in Fig. 5, where a band covers the performances of all the models in each group and a line shows the median performance of each model group.

Considering both the training and testing phases, $\mathbf {T_{Grid-CNN}}$ models are more easily to overfit the training data (a high training PC - median of 0.9899, but moderate testing PCs - medians of 0.6128/0.7090 for the two CSAR-HiQ sets). In the testing phase, $\mathbf {T_{IMC-CNN}}$ and $\mathbf {T_{Graph-GCN}}$ models stand out as two strong competitors (median testing PCs of 0.6396/0.6847 for $\mathbf {T_{IMC-CNN}}$ and 0.6424/0.7054 for $\mathbf {T_{Graph-GCN}}$), while $\mathbf {T_{ACNN}}$ models generally perform inadequately in the predictions (median testing PCs of 0.5363/0.6785). The $\mathbf {T_{Grid-CNN}}$ models have a wider span in PC, mostly because of the marked difference between augmented grids and original data. However, the large computational resources consumed in the learning of augmented data by $\mathbf {T_{Grid-CNN}}$ models strongly hinder the further development of such models. As shown in our experiments, quadrupled grids led to an approximately four-time growth in training time and storing memory (Additional file1: Table S3). Taking into account the prediction accuracy and required computational resources, $\mathbf {T_{Graph-GCN}}$ models are arguably the most promising and refinable methods in current PLBAP tasks.

Regarding the 26 representative models, the best performers in terms of the validation PC ($M_5$, $M_9$, $M_{12}$ and $M_{26}$ in Additional file 1: Table S2) were selected to stand for the four types of models. These models are described as follows.

- $M_5$ is a $T_{ACNN}$ model. It employs 12 neighbors and 15 atom types in the atom-type convolution layer. A distance threshold of $R_c=12\mathring{A}$, 6 filters (interval of $2\mathring{A}$ for $r_q$) and $\sigma _q^2=2.5$ are adopted for radial pooling. 3 atomistic dense layers (sizes of 32, 32 and 16) are stacked to yield the molecular energy. The whole model was trained with 200 epochs and a batch size of 24.
- $M_9$ is a $T_{IMC-CNN}$ model. Its feature representation ($64\times 60$ matrix) concerns 64 IMCs and 60 distance shells (from **OnionNet**). The model, with a similar architecture as OnionNet ($conv1 = 16$, $conv2 = 64$ and $conv3 = 128$), was trained with 200 epochs and a batch size of 128.
- $M_{12}$ is a $T_{Grid-CNN}$ model. Its feature representation ($21\times 21\times 21\times 16$ tensor) emphasizes a $20\mathring{A}\times 20\mathring{A}\times 20\mathring{A}$ grid with a resolution of $1\mathring{A}$, and captures the properties of protein and ligand atoms separately (each for 8 properties from **KDEEP**) at each voxel. The final model, with a light-weight architecture from **KDEEP**, was trained with 100 epochs, a batch size of 64 and a learning rate of $10^{-5}$ (*L*2-regularization adopted to prevent from overfitting).
- $M_{26}$ is a $T_{Graph-GCN}$ model. A threshold of $6\mathring{A}$, which crops a binding area of $<400$ atoms for each complex, is adopted by this model. Its feature representation then involves a node-feature matrix ($400\times 18$) concerning 18 atomic properties from **Pafnucy**, and an adjacency tensor ($400\times 400\times 3$) with each slice indicating intermolecular contacts in a certain range ($0\sim 2\mathring{A}$, $2\mathring{A}\sim 4\mathring{A}$ or $4\mathring{A}\sim 6\mathring{A}$). The model, with a similar architecture as **GraphBAR** (4 layers in each convolutional block), was trained with 200 epochs and a batch size of 64.

The scoring performances of these models are exhibited in Table 2.

**Table 2:** Representative deep-learning PLBAP models and their scoring performances

Model (ID)	Training (*PDBbind Refined Set*)		Parameter-tuning (*PDBbind Core Set*)		Test1 (*CSAR-HiQ Set 1*)		Test2 (*CSAR-HiQ Set 2*)	
	PC	RMSE	PC	RMSE	PC	RMSE	PC	RMSE
$\mathbf {T_{ACNN}}$ ($M_5$)	0.5189	1.7564	0.5692	1.7939	0.5596	1.9749	0.6804	1.6298
$\mathbf {T_{IMC-CNN}}$ ($M_9$)	0.7851	1.2607	0.7843	1.4807	0.6365	1.8011	0.6123	1.7329
$\mathbf {T_{Grid-CNN}}$ ($M_{12}$)	0.9224	0.8939	0.9235	1.0079	0.5531	1.9451	0.684	1.6373
$\mathbf {T_{Graph-GCN}}$ ($M_{26}$)	0.6403	1.5178	0.6969	1.6733	0.6706	1.7414	0.737	1.5098

### Model interpretability

**Fig. 6:**
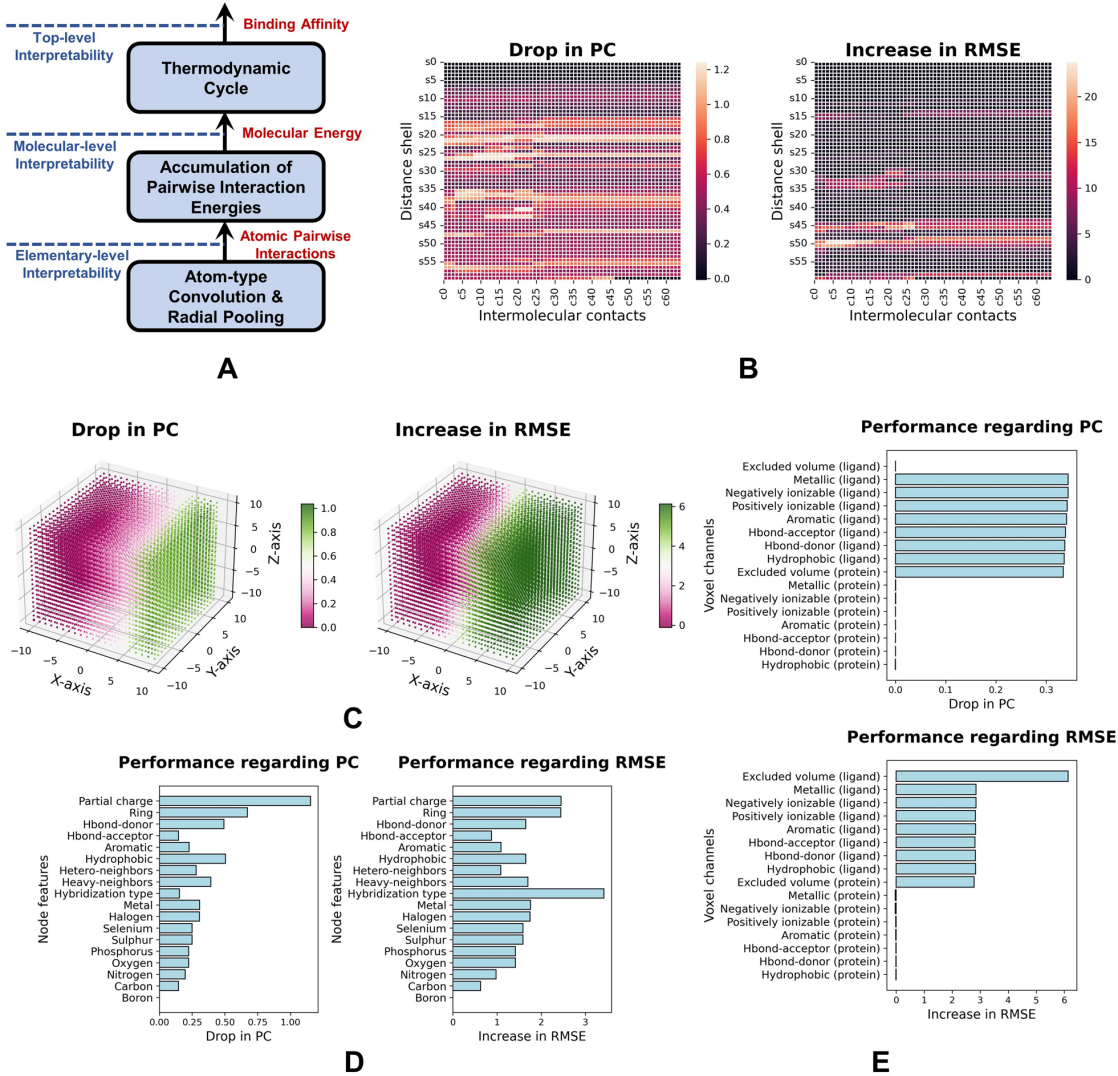
Interpretability of representative PLBAP models. **A** Model-level interpretability of $T_{ACNN}$ models. **B** Heatmaps showing the feature importance for a $T_{IMC-CNN}$ model. **C** Heatmaps showing the importance of position-related features for a $T_{Grid-CNN}$ model. **D** Importance of the node features for a $T_{Graph-GCN}$ model. **E** Importance of the voxel channels for a $T_{Grid-CNN}$ model

$T_{ACNN}$ models can be explained, to some extent, at the model-level (Fig. 6A). While the other three types of models ($T_{IMC-CNN}$, $T_{Grid-CNN}$ and $T_{Graph-GCN}$) can be interpreted in a post-hoc manner, mostly by revealing the feature significance and detecting hot-spot areas. Based on the three best performers in Table 2 ($M_9$ for $T_{IMC-CNN}$, $M_{12}$ for $T_{Grid-CNN}$ and $M_{26}$ for $T_{Graph-GCN}$), we leveraged a dataset-level masking technique to uncover important features for each model. We first evaluated each model on the validation set (*PDBbind Core Set*), yielding the PC of $pc_0$ and the RMSE of $rmse_0$. Then specific features were masked (set to zero) for all complexes in the validation set, and the masked data were fed into the model for a re-evaluation (yielding $pc_i$ and $rmse_i$). A larger PC drop ($\Delta pc_i = pc_i-pc_0$) or RMSE increase ($\Delta rmse_i = rmse_i-rmse_0$) implies higher importance of the masked features.

$\mathbf {T_{IMC-CNN}}$. $M_9$ represents a complex by an IMC matrix ($64\times 60$), where each position (*j*, *k*) in this matrix is a specific feature and its importance can be measured through the masking scheme. By collecting the importance data with respect to all the positions, the heatmaps regarding PC drops and RMSE increases were generated (Fig. 6B). Here intermolecular contacts in distance shells $s_{20}\sim s_{26}$ ($11\mathring{A}\sim 14\mathring{A}$) are more highlighted for a PC drop, and those in $s_{44}\sim s_{52}$ ($23\mathring{A}\sim 27\mathring{A}$) are more important for an RMSE increase. Another model $M_7$ in this category can be explained similarly, as displayed in Additional file 1: Figure S1. $\mathbf {T_{Grid-CNN}}$. $M_{12}$ characterizes a complex by a molecular grid ($21\times 21\times 21\times 16$), and we masked the features in two ways. First, each position (*j*, *k*, *l*) ($1\le j,k,l\le 21$) in the grid was masked for importance investigation (Fig. 6C). Here the origin is the ligand center and the protein atoms around this center show higher importance in PC drops or RMSE increases. Due to the various protein-ligand binding orientations, this dataset-level study can only show a rough picture of the position importance. Second, we masked each property channel of the grid voxels (total of 16 channels), leading to an importance plot in Fig. 6E. Apparently, the ligand-related channels play a more important role than the protein-related channels, and the increase in RMSE is more correlated with the excluded volume of ligand atoms. A similar interpretation for $M_{11}$ in this category is shown in Additional file 1: Figures S2$\sim$3. $\mathbf {T_{Graph-GCN}}$. $M_{26}$ represents a complex by a node-feature matrix ($400\times 18$) and an adjacency tensor ($400\times 400\times 3$). Each node feature (total of 18 features) was examined according to the masking technique, generating an importance plot in Fig. 6D. As shown here, features like partial charge, ring membership, hydrophobicity and hydrogen-bond donor are more important for a PC drop. The hybridization type stands out for an increase in RMSE, followed by partial charge and ring membership. As another example, $M_{23}$ in this category can be interpreted by Additional file 1: Figure S4.

### Evaluation of screening performances

As another evaluation of above models, the screening powers that show the capability of identifying active binders (actives) from non-binders (decoys) were estimated. ***Validation data.*** As a frequently-accessed database in molecular docking tasks, the enhanced directory of useful decoys (DUD-E) provides challenging decoys to active compounds binding to specific target proteins. Two targets, muscle glycogen phosphorylase (PYGM) and epidermal growth factor receptor (EGFR), from DUD-E were considered. PYGM concerns 114 actives and 4045 decoys, leading to a small set of 4159 PYGM-ligand pairs. EGFR has 832 actives and 35,441 decoys, constituting a large set of 36,273 EGFR-ligand pairs. These two sets (details in Additional file 1 : Table S1) were used to contrastively investigate the screening powers of the deep-learning PLBAP models. The decoy-to-active ratios ($r_{DTA}=\frac{n_{decoy}}{n_{active}}$) of these two sets are approximately 35.5 and 42.6. ***Generating protein-ligand complexes.*** Due to the lack of complex structures, the data in DUD-E could not be fed into deep-learning BAP models directly. As such, AutoDOCK Vina was leveraged to generate the protein-ligand complex structures (binding poses), each with a docking grid of $20\mathring{A}\times 20\mathring{A}\times 20\mathring{A}$ placed at the ligand-center position of the template structure (PDB:1C8K for PYGM-ligand pairs and PDB:2RGP for EGFR-ligand pairs). When docking each pair of molecules using *Vina*, 32 consecutive Monte-Carlo samplings were conducted and the best pose was outputted during the search. These parameters are commonly adopted in docking applications. ***Evaluation rules.*** Relying on a deep-learning PLBAP model, the binding affinities for target-ligand complexes can be predicted and ranked. The proportion of actives in the top $X\%$ of ranked ligands, namely the enrichment factor ($EF^{X}$), is a crucial indicator showing the screening power of the model. Given an $r_{DTA}$ ($1,2,\ldots ,r_{DTA}^{max}$), the decoys can be randomly selected from the decoy pool, and we can calculate $EF^{X}$ for the actives coupled with selected decoys. The top $1\sim 5\%$ of ranked ligands ($X=1,2,\ldots ,5$) were investigated in the enrichment analysis. To prevent from randomness, 10 selections were drawn and averaged to produce the final $EF^{X}$ for each $r_{DTA}$ and *X* values. A higher $EF^{X}$ normally indicates a better screening performance.

**Fig. 7:**
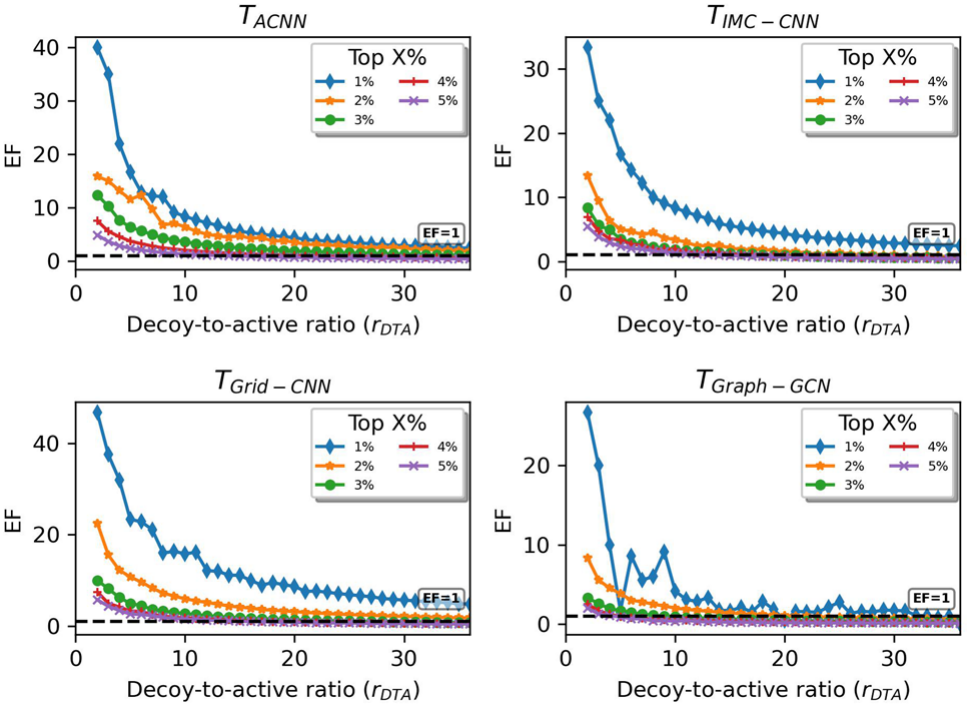
Screening performances of representative deep-learning PLBAP models on PYGM dataset from *DUD-E*

The enrichment analysis was conducted to reveal the screening powers of PLBAP models on the PYGM and EGFR datasets (Figs. 7$\sim$8). Here $M_5$, $M_9$ and $M_{26}$ (Table 2) were selected to stand for $T_{ACNN}$, $T_{IMC-CNN}$ and $T_{Graph-GCN}$ models. Since $T_{Grid-CNN}$ models have a severer overfitting problem (as shown in Table 2), we adopted model $M_{14}$, which is computationally more expensive (built on augmented data) but with a better testing performance (Additional file 1 : Tables S2$\sim$3), to represent $T_{Grid-CNN}$. Generally speaking for Figs. 7$\sim$8, as $r_{DTA}$ increases, $EF^X$ decreases dramatically. The real applications often involve a high $r_{DTA}$ as actives are always the minority in the broad compound space, which puts a major obstacle to current PLBAP works. For the small PYGM dataset, the $T_{Grid-CNN}$ model performs marginally better as $r_{DTA}$ increases, particularly for the top $1\%$ complexes. For the larger EGFR set that is more similar to the real states, $T_{Graph-GCN}$ and $T_{IMC-CNN}$ models are more competitive. Especially, the $T_{Graph-GCN}$ model retains an *EF* of $10\sim 20$ as $r_{DTA}$ reaches 40, for the top $1\%$ complexes. As such, $T_{Graph-GCN}$ models have better potential to be developed into more powerful screening machines.

**Fig. 8:**
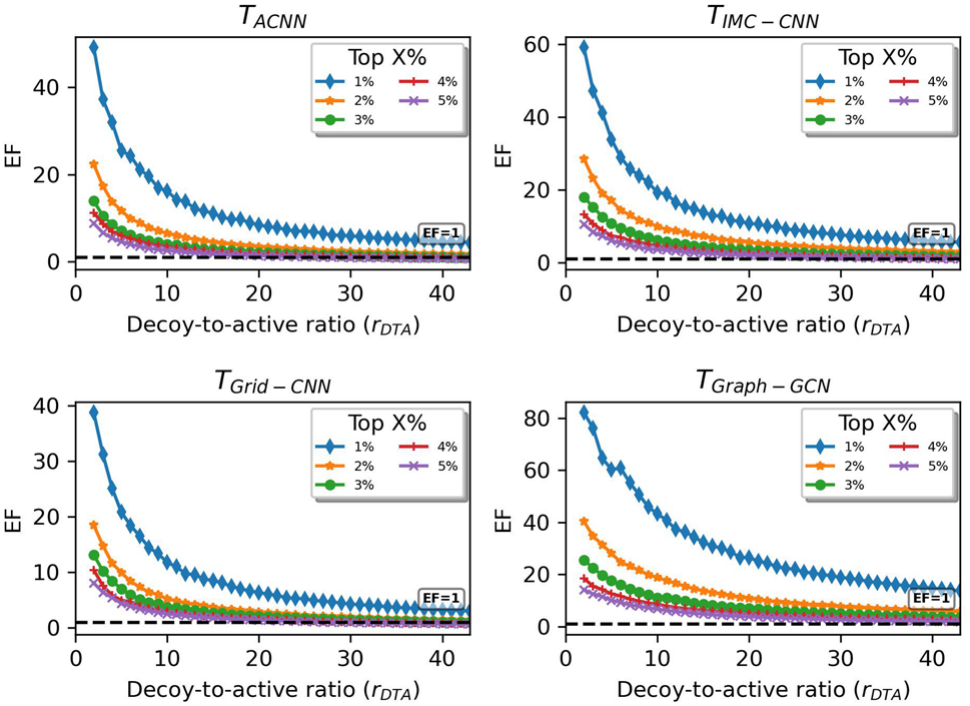
Screening performances of representative deep-learning PLBAP models on EGFR dataset from *DUD-E*

## Conclusions

Deep-learning PLBAP models have their pros and cons that need to be weighted up for specific scoring tasks. $\mathbf {T_{ACNN}}$ models can be explained from the perspective of energy and thermodynamic cycle, and it is friendly to large-scale computations. However, they often have insufficient learning abilities for scoring or screening tasks. $\mathbf {T_{IMC-CNN}}$ models count on the learning of multi-range intermolecular contact features by 2D-CNN models. The feature representations are simple and can be efficiently learned. But such representations oversimplify the protein-ligand interactions and ignore the spatial information of the molecules, making the explanation from the structural and physico-chemical perspectives more difficult. $\mathbf {T_{Grid-CNN}}$ models leverage the molecular structural information and voxelization techniques, laying a foundation of structural interpretation of protein-ligand interactions. But the generation of such voxel features is resource-intensive, rendering the generalization to large-scale computations impractical. The lack of rotational invariance puts even more obstacles to such models, particularly in screening tasks. $\mathbf {T_{Graph-GCN}}$ models have demonstrated great potential recently. They are less resource-intensive but can capture molecular topologies more flexibly than $\mathbf {T_{Grid-CNN}}$ models, making them competitive in scoring and screening tasks. Refining the graph representations, developing neat but powerful learning architectures, and enhancing the interpretability can be promising ways to explore the potential of such models deeply. Devising more powerful machines, which are accurate in scoring tasks and also robust to tough screening tasks (with high $r_{DTA}$), will be a key direction for future developments of PLBAP works.

### Supplementary Information

**Additional file 1: Table S1.** Description about the datasets in this study. **Table S2.** Scoring performances of deep-learning PLBAP models. **Table S3.** Training times of some good-performing PLBAP models. To make a fair comparison, a 20-trial random search for hyperparameter tuning was adopted for each model to yield the time costs. The higher time costs for each type of models are highlighted. **Figure S1.** Heatmaps showing the importance of features, in terms of PC drop and RMSE increase, for M_7_ model. These features concern 30 distance shells (s0 ∼ s29) and 36 types of intermolecular contacts (c0 ∼ c35). **Figure S2.** Heatmaps showing the importance of positions, in terms of PC drop and RMSE increase, for M_11_ model. Each position is a voxel, characterized by 9 channels (hydrophobicity, hydrogen-bond donor, hydrogen-bond acceptor, aromaticity, positivelyionizable, negatively ionizable, metallicity, excluded volume, and sign for a protein/ligand atom). **Figure S3.** Importance of voxel channels, in terms of PC drop and RMSE increase, for M_11_ model. **Figure S4.** Importance of node features, in terms of PC drop and RMSE increase, for M_23_ model.

## Data Availability

The data for PLBAP-model construction (training and hyperparameter-tuning) are from the *PDBbind* database (http://www.pdbbind.org.cn/). The test sets for evaluating the scoring performances of constructed models stem from *CASR* (http://csardock.org/). The screening powers of those models were measured using the PYGM and EGFR targets from *DUD-E* (https://dude.docking.org/). The coding and experiment guidelines can be found from the online GitHub repository (https://github.com/debbydanwang/DL-PLBAP).

## Abbreviations

CDD: Computational drug discovery
RMSD: Root-mean-square deviation
PLBAP: Protein-ligand binding affinity prediction
DNN: Deep neural network
ACNN: Atomic convolutional neural network
2D-CNN: Two-dimensional convolutional neural network
3D-CNN: Three-dimensional convolutional neural network
GCN: Graph convolutional network
GGNN: Gated graph neural network
IMC: Intermolecular contact
IMCP: Intermolecular contact profile
PC: Pearson’s correlation
RMSE: Root-mean-squared error
DUD-E: Enhanced directory of useful decoys
PYGM: Muscle glycogen phosphorylase
EGFR: Epidermal growth factor receptor
