# Supplementary material for: Structure-based, deep-learning models for protein-ligand binding affinity prediction
Source: J Cheminform. 2024 Jan 3;16:2. doi: 10.1186/s13321-023-00795-9 (PMC10765576; doi:10.1186/s13321-023-00795-9)
Supplement: Supplementary file 1 — Additional file 1: Table S1. Description about the datasets in this study. Table S2. Scoring performances of deep-learning PLBAP models. Table S3. Training times of some good-performing PLBAP models. To make a fair comparison, a 20-trial random search for hyperparameter tuning was adopted for each model to yield the time costs. The higher time costs for each type of models are highlighted. Figure S1. Heatmaps showing the importance of features, in terms of PC drop and RMSE increase, for M7 model. These features concern 30 distance shells (s0 ∼ s29) and 36 types of intermolecular contacts (c0 ∼ c35). Figure S2. Heatmaps showing the importance of positions, in terms of PC drop and RMSE increase, for M11 model. Each position is a voxel, characterized by 9 channels (hydrophobicity, hydrogen-bond donor, hydrogen-bond acceptor, aromaticity, positivelyionizable, negatively ionizable, metallicity, excluded volume, and sign for a protein/ligand atom). Figure S3. Importance of voxel channels, in terms of PC drop and RMSE increase, for M11 model. Figure S4. Importance of node features, in terms of PC drop and RMSE increase, for M23 model. [file 13321_2023_795_MOESM1_ESM.pdf]

Structure-based, Deep-learning Models for Protein-ligand Binding Affinity Prediction

Debby D. Wang[1], Wenhui Wu[2] and Ran Wang[3,*]

[1]School of Science and Technology, Hong Kong Metropolitan University, Kowloon, Hong Kong
[2]College of Electronics and Information Engineering, Shenzhen University, Shenzhen 518060, China
[3]College of Mathematics and Statistics, Shenzhen University, Shenzhen 518060, China
[*]Correspondence author: Ran Wang, E-mail: wangran@szu.edu.cn

1 Description about Datasets

Three data sources (*PDBbind*, *CSAR* and *DUD-E*) were considered to investigate the performances of different DLBAP models. Each model was trained on the *PDBbind Refined Set*, with the *PDBbind Core Set* used for hyperparameter tuning. These models were then tested on two sets from CSAR, namely *CSAR-HiQ Set 1* and *CSAR-HiQ Set 2*, to yield their scoring performances. To further examine the screening performances of PLBAP models, the *PYGM* and *EGFR* sets from *DUD-E* were adopted. Details of these sets were tabulated in Supplementary Table 1.

Additional file 1: Table S1: Description about the datasets in this study.

Set	Origin	Size	Size after redundancy removal	Purpose of use
Refined Set	*PDBbind* (http://www.pdbbind.org.cn/)	5,316 binding complexes	4,856 binding complexes	PLBAP-model training
Core Set	*PDBbind* (http://www.pdbbind.org.cn/)	285 binding complexes	285 binding complexes	PLBAP-model hyperparameter tuning
CSAR-HiQ Set 1	*CSAR* (http://www.csardock.org/)	175 binding complexes	175 binding complexes	Testing the scoring performances of PLBAP models
CSAR-HiQ Set 2	*CSAR* (http://www.csardock.org/)	167 binding complexes	167 binding complexes	Testing the scoring performances of PLBAP models
PYGM Set	*DUD-E* (https://dude.docking.org/)	4,159 protein-ligand pairs (114 actives / 4045 decoys)	4,159 protein-ligand pairs (114 actives / 4045 decoys)	Investigating the screening performances of PLBAP models
EGFR Set	*DUD-E* (https://dude.docking.org/)	36,273 protein-ligand pairs (832 actives / 35,441 decoys)	36,273 protein-ligand pairs (832 actives / 35,441 decoys)	Investigating the screening performances of PLBAP models

2 Model Construction Details

By altering the feature representations and model architectures, 26 representatives ($M_1 \sim M_{26}$) belonging to the four types of models were constructed. Each model, with a specific feature representation and a learning architecture, was trained with parameters (e.g. batch size and number of epochs) tuned in the training process. The model construction and training details are elaborated as follows.

$\mathbf{T_{ACNN}}$ Models. When realizing the **ACNN** model, a fixed number of atoms for the ligand ($N_L = 70$), protein ($N_P = 630$) or complex ($N_{PL} = 700$) was adopted for batch calculations. These numbers cover sufficient atoms in the protein-ligand binding pocket. Insufficient atoms in a ligand (e.g. < 70) were zero-padded. As adopted by the original work, 12 neighbors and 15 atom types (C, N, O, F, Na, Mg, P, S, Cl, Ca, Mn, Zn, Br, I and others) were employed in the atom-type convolution layer. For radial pooling with a distance threshold of $R_c = 12\mathring{A}$, different numbers of filters Q were investigated (3, 4, or 6). These Q filters each had their $r_q = (q-1) \times \frac{12}{Q}$ and a fixed σ_q^2 (2.5 or 1). 3 atomistic dense layers (sizes of 32, 32 and 16) were stacked to yield the estimated energy of each input molecular fragment. Although the original **ACNN** work used a fixed batch size (24) and number of epochs (100), the batch size (10, 20 or 24) and training epochs (50, 100, 150 or 200) were tuned via a 15-trial random search in this work. The best-performing model was retrained on the combined training and validation data (with one epoch) to be the final model, which was tested on the CSAR-HiQ data sets.

$\mathbf{T_{IMC-CNN}}$ Models. Two types of IMC-shell matrices were employed. One is inspired by **OnionNet** and concerns 64 IMCs in 60 distance shells, leading to a 64×60 matrix for each protein-ligand complex. The other one involves 36 IMCs from **RF-Score** in 30 distance shells ($0\mathring{A} \sim 30\mathring{A}$ at an interval of $1\mathring{A}$). Above two types of feature representations only consider the quantities of IMCs, and adding the average atomic distances results in another two IMCP representations (size of $64 \times 60 \times 2$ and $36 \times 30 \times 2$ for each complex). 2D-CNNs with a classic architecture (used by **OnionNet**) were adopted to learn the matrix features and build PLBAP models. Although **OnionNet** uses fixed numbers of filters in three consecutive convolutional layers (*conv1*: 32, *conv2*: 64 and *conv3*: 128), we slightly tuned them in this work (*conv1*: 16, 24 or 32, *conv2*: 32, 48 or 64, *conv3*: 64, 96 or 128). Commonly-used training epochs (50, 100, 150 or 200) and batch sizes (5, 64 or 128) were also tuned. Such hyperparameter tunning was benefitted from a 350-trail random search by *KerasTuner*. The best performer, with respect to feature representation and learning model, was then retrained on the combined training and validation data, and evaluated ultimately on the CSAR-HiQ data sets.

$\mathbf{T_{Grid-CNN}}$ Models. As a prevalent setting, the 3D-grid area was standardized as a $20\mathring{A} \times 20\mathring{A} \times 20\mathring{A}$ box having a coincided center with the ligand and a resolution of $1\mathring{A}$. The 8 atom-level features (hydrophobicity, aromaticity, H-bond donor/acceptor, positive/negative charge, metallicity and excluded volume) adopted by **KDEEP** were the base for generating grid representations, and a simple aggregation strategy was used to fill each grid by these features. A filled grid that covers both protein and ligand atoms led to a $21 \times 21 \times 21 \times 8$ tensor for a complex, while filling two grids separately for the protein and ligand resulted in a $21 \times 21 \times 21 \times 16$ tensor. To compensate the lack of rotational invariance, rotating a tensor will generate new samples and therefore augment such data. In this work, the $21 \times 21 \times 21 \times 16$ tensors were quadrupled by rotating the Euler angles ($\alpha \in [0, 2\pi]$, $\beta \in [0, \pi]$ and $\gamma \in [0, 2\pi]$) of each protein-ligand complex system. As an example, four rotations corresponding to Euler angles of $(0, 0, 0)$, $(\pi, 0, 0)$, $(0, \frac{\pi}{2}, 0)$ and $(\pi, \frac{\pi}{2}, 0)$ were conducted if α and β were emphasized. The augmented samples have the same label (binding affinity) with the original sample. To investigate different atom-level features, a simple set of atom types in **RF-Score** (C, N, O and S for protein atoms, and C, N, O, F, P, S, Cl, Br and I for ligand atoms) were adopted for generating the 3D-grids ($21 \times 21 \times 21 \times 13$), and such tensors were similarly augmented as above. Due to the resource-intensive nature of grid representations, the light-weight 3D-CNN architecture from **KDEEP** was employed in our model training. $L2$-regularization (with a default λ_{L2} of 0.01) was used to prevent from overfitting. To properly and efficiently learn the resource-intensive features, the learning rate (0.0001, 0.00001 or 0.000001), training epochs (50 or 100) and batch sizes (5, 64 or 128) were tuned by a *KerasTuner* random search with 20 trials. According to the experiments, $\mathbf{T_{Grid-CNN}}$ models are more easily to overfit the training data, and therefore less training epochs than the other models are preferred. Finally, the models were retrained, and tested on the CSAR-HiQ data. The prediction values for the same system (original or rotated) were averaged to indicate its final binding affinity, and this averaged value was adopted for yielding the PC and RMSE.

$\mathbf{T_{Graph-GCN}}$ Models. When generating the molecular graphs, we investigated a number of contributors to such representations. First, the binding site of each complex was defined as those atoms within a distance threshold of any ligand atom. **GraphBAR** adopts a threshold of $4\mathring{A}$ and a maximum number of 200 atoms. In addition to this setting, thresholds of $6\mathring{A}$ and 400 atoms were also examined. Second, for each node (atom) in a molecular graph, we employed two sets of features (8 features from **KDEEP** or 18 features from **Pafnucy**) to characterize the nodes. As graph representations are not that resource-intensive as grid representations, involving more informative features can be a feasible way to describe the nodes better. Third, two strategies to measure the adjacencies in the molecular graphs were considered. One strategy leverages two adjacency matrices to account for the covalent bonds (binary) and distance adjacencies (continuous). The other strategy measures atomic adjacencies by fixed-interval distance shells (2 shells for a binding site with $4\mathring{A}$-cutoff and 3 shells for that with $6\mathring{A}$-cutoff) and adopts binary adjacency matrices in the representations. Finally, the learning architecture from **GraphBAR** was employed. Instead of using a fixed parameter setting, the number of layers in graph convolutional blocks (3, 4 or 5), training epochs (50, 100, 150 or 200) and batch size (5, 64 or 128) were tuned via a 40-trial random search from *KerasTuner*. The best performers were tested on the CSAR-HiQ data sets.

3 Model Evaluation Results

All experiments were GPU-accelerated (NVIDIA Tesla V100 with a 32GB RAM). The scoring performances of the 26 models are displayed in Supplementary Table 2. The training times for some descent performers (with respect to validation PC) were also examined. To provide a fair comparison, the training time with a uniform setting (20-trial random search for hyperparameter tuning) for each model was recorded, as shown in Additional file 1: Table S3.

Supplementary Table 2: Scoring performances of deep-learning PLBAP models.

Model Type	ID	Feature Representation	Hyperparameters (Tuned)+	Other Parameters+	Training*		Validation*		Test1*		Test2*	
					PC	RMSE	PC	RMSE	PC	RMSE	PC	RMSE
$\mathbf{T_{ACNN}}$	M_1	Atom Coordinates: 700×3 (complex), 630×3 (protein), 70×3 (ligand) Atom Types: 700×1 (complex), 630×1 (protein), 70×1 (ligand)	$epc=150$, $bs=24$	$Q=3$, $\sigma_q^2=2.5$	0.5266	1.6598	0.5662	1.7996	0.5445	1.9232	0.6766	1.6132
	M_2		$epc=100$, $bs=20$	$Q=3$, $\sigma_q^2=1$	0.5237	1.7982	0.5448	2.0117	0.5281	2.1474	0.6826	1.8333
	M_3		$epc=150$, $bs=20$	$Q=4$, $\sigma_q^2=2.5$	0.5291	1.703	0.55	1.9104	0.5702	1.983	0.6813	1.7086
	M_4		$epc=100$, $bs=24$	$Q=4$, $\sigma_q^2=1$	0.4817	3.1449	0.4967	3.1633	0.4778	3.2779	0.6293	3.2184
	$M_5\star$		$epc=200$, $bs=24$	$Q=6$, $\sigma_q^2=2.5$	0.5189	1.7564	0.5692	1.7939	0.5596	1.9749	0.6804	1.6298
	M_6		$epc=200$, $bs=128$	$Q=6$, $\sigma_q^2=1$	0.5584	1.7523	0.5237	1.9062	0.4976	2.0486	0.6755	1.6562
$\mathbf{T_{IMC-CNN}}$	M_7	36×30 matrices	$conv1=16$, $conv2=64$, $conv3=96$, $epc=200$, $bs=128$	–	0.7404	1.3046	0.7515	1.4508	0.703	1.6282	0.6903	1.5698
	M_8	$36\times30\times2$ tensors	$conv1=32$, $conv2=64$, $conv3=96$, $epc=200$, $bs=128$	–	0.6526	1.487	0.6558	1.6854	0.6427	1.7607	0.7167	1.5778
	$M_9\star$	64×60 matrices	$conv1=16$, $conv2=64$, $conv3=128$, $epc=200$, $bs=128$	–	0.7851	1.2607	0.7843	1.4807	0.6365	1.8011	0.6123	1.7329
	M_{10}	$64\times60\times2$ tensors	$conv1=32$, $conv2=64$, $conv3=64$, $epc=200$, $bs=128$	–	0.5938	1.5847	0.6767	1.7789	0.589	1.8937	0.6791	1.6518
$\mathbf{T_{Grid-CNN}}$	M_{11}	$21\times21\times21\times8$ tensors	$lr=0.00001$, $epc=100$, $bs=64$	$\lambda_{L2}=0.01$	0.9856	0.4241	0.8543	1.1787	0.4017	2.212	0.4656	2.0746
	$M_{12}\star$	$21\times21\times21\times16$ tensors	$lr=0.00001$, $epc=100$, $bs=64$	$\lambda_{L2}=0.01$	0.9224	0.8939	0.9235	1.0079	0.5531	1.9451	0.684	1.6373
	M_{13}	$21\times21\times21\times16$ tensors (quadrupled[1])	$lr=0.00001$, $epc=100$, $bs=64$	$\lambda_{L2}=0.01$	0.9858	0.9039	0.7381	1.7125	0.5992	1.9929	0.6431	1.8168
	M_{14}	$21\times21\times21\times16$ tensors (quadrupled[2])	$lr=0.00001$, $epc=100$, $bs=64$	$\lambda_{L2}=0.01$	0.9137	1.0243	0.8887	1.1773	0.604	1.9077	0.7169	1.6534
	M_{15}	$21\times21\times21\times16$ tensors (quadrupled[3])	$lr=0.00001$, $epc=100$, $bs=64$	$\lambda_{L2}=0.01$	0.917	1.0698	0.8711	1.2899	0.603	1.9347	0.7188	1.6747
	M_{16}	$21\times21\times21\times13$ tensors (quadrupled[1])	$lr=0.00001$, $epc=100$, $bs=64$	$\lambda_{L2}=0.01$	0.9951	0.1935	0.7973	1.3183	0.6794	1.6754	0.7339	1.4749
	M_{17}	$21\times21\times21\times13$ tensors (quadrupled[2])	$lr=0.00001$, $epc=100$, $bs=64$	$\lambda_{L2}=0.01$	0.994	0.3013	0.8228	1.2592	0.6466	1.7745	0.7011	1.5581
	M_{18}	$21\times21\times21\times13$ tensors (quadrupled[3])	$lr=0.00001$, $epc=100$, $bs=64$	$\lambda_{L2}=0.01$	0.9945	0.4491	0.7916	1.4184	0.6215	1.8608	0.6809	1.6518
$\mathbf{T_{Graph-GCN}}$	M_{19}	$\mathcal{M}_V$: 200×8 matrices, $\mathbf{A}$: $200\times200\times2$ tensors[4]	$L_{GCB}=4$, $epc=150$, $bs=5$	–	0.5511	1.625	0.6334	1.7777	0.6214	1.8311	0.6878	1.6371
	M_{20}	$\mathcal{M}_V$: 400×8 matrices, $\mathbf{A}$: $400\times400\times2$ tensors[4]	$L_{GCB}=4$, $epc=100$, $bs=5$	–	0.4834	1.7015	0.5821	1.8633	0.5504	1.9302	0.677	1.6936
	M_{21}	$\mathcal{M}_V$: 200×8 matrices, $\mathbf{A}$: $200\times200\times2$ tensors[5]	$L_{GCB}=4$, $epc=100$, $bs=5$	–	0.532	1.6645	0.6456	1.7989	0.6396	1.8439	0.702	1.6432
	M_{22}	$\mathcal{M}_V$: 400×8 matrices, $\mathbf{A}$: $400\times400\times3$ tensors[6]	$L_{GCB}=3$, $epc=100$, $bs=5$	–	0.5167	1.6611	0.6108	1.7903	0.5963	1.8557	0.6931	1.6335
	M_{23}	$\mathcal{M}_V$: 200×18 matrices, $\mathbf{A}$: $200\times200\times2$ tensors[4]	$L_{GCB}=3$, $epc=200$, $bs=128$	–	0.6497	1.5352	0.6526	1.7452	0.6451	1.7969	0.7089	1.6396
	M_{24}	$\mathcal{M}_V$: 400×18 matrices, $\mathbf{A}$: $400\times400\times2$ tensors[4]	$L_{GCB}=3$, $epc=200$, $bs=128$	–	0.6096	1.6945	0.6611	1.7326	0.6478	1.824	0.7109	1.6763
	M_{25}	$\mathcal{M}_V$: 200×18 matrices, $\mathbf{A}$: $200\times200\times2$ tensors[5]	$L_{GCB}=3$, $epc=200$, $bs=128$	–	0.6446	1.5267	0.625	1.7368	0.6536	1.7385	0.7127	1.5784
	$M_{26}\star$	$\mathcal{M}_V$: 400×18 matrices, $\mathbf{A}$: $400\times400\times3$ tensors[6]	$L_{GCB}=4$, $epc=200$, $bs=64$	–	0.6403	1.5178	0.6969	1.6733	0.6706	1.7414	0.737	1.5098

* Trained on PDBbind *Refined Set* and validated on the *Core Set* (for hyperparameter tuning). Test sets are the CSAR-HiQ sets.

\+ epc: number of epochs, bs: batch size, $conv1\sim3$: number of filters in the convolutional layers, lr: learning rate, λ_{L2}: L2-regularization rate, L_{GCB}: number of layers in a graph convolutional block.

$\star$: best validation PC in each group of models.

1 Augmenting the grid data by rotating the α and β Euler angles.

2 Augmenting the grid data by rotating the α and γ Euler angles.

3 Augmenting the grid data by rotating the β and γ Euler angles.

4 Considering covalent bonds (binary matrix) and distance adjacencies (distance matrix).

5 Considering two binary adjacency matrices accounting for atomic contacts within distance shells of $(0, 2\AA]$ and $(2\AA, 4\AA]$.

6 Considering three binary adjacency matrices accounting for atomic contacts within distance shells of $(0, 2\AA]$, $(2\AA, 4\AA]$ and $(4\AA, 6\AA]$.

Additional file 1: Table S3: Training times of some good performing PLBAP models. To make a fair comparison, a 20-trial random search for hyperparameter tuning was adopted for each model to yield the time costs. The higher time costs for each type of models are highlighted.

Model (ID)	Training Time (min)
$\mathbf{T}_{\mathbf{ACNN}}$ (M_1)	61.23
$\mathbf{T}_{\mathbf{ACNN}}$ (M_5)	***65.04***
$\mathbf{T}_{\mathbf{IMC-CNN}}$ (M_7)	121.53
$\mathbf{T}_{\mathbf{IMC-CNN}}$ (M_9)	***320.20***
$\mathbf{T}_{\mathbf{Grid-CNN}}$ (M_{11})	249.91
$\mathbf{T}_{\mathbf{Grid-CNN}}$ (M_{12})	256.26
$\mathbf{T}_{\mathbf{Grid-CNN}}$ (M_{14})	***1015.66***
$\mathbf{T}_{\mathbf{Graph-GCN}}$ (M_{23})	114.32
$\mathbf{T}_{\mathbf{Graph-GCN}}$ (M_{26})	***214.24***

4 Post-hoc Interpretability of DLBAP Models

$T_{IMC-CNN}$, $T_{Grid-CNN}$ and $T_{Graph-GCN}$ models (M_9, M_{12} and M_{26}) were interpreted from a feature-importance perspective, depending on a dataset-level masking technique. The feature-importance plots for these models are separately exhibited in Supplementary Figures 1~4.

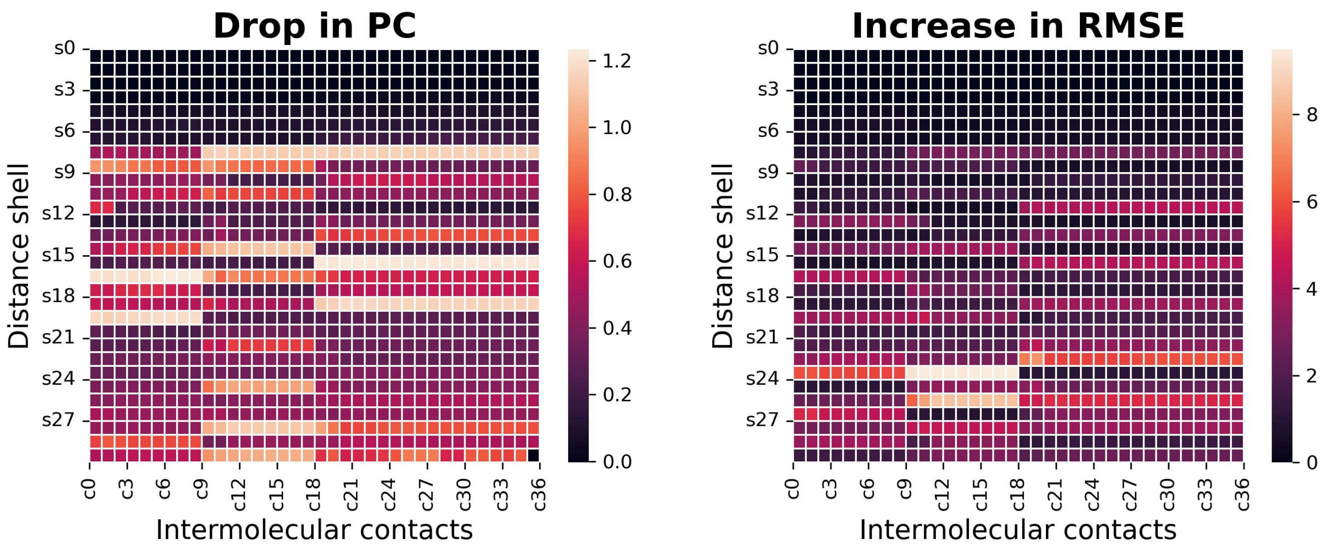

Additional file 1 Figure S1: Heatmaps showing the importance of features, in terms of PC drop and RMSE increase, for M_7 model. These features concern 30 distance shells ($s0 \sim s29$) and 36 types of intermolecular contacts ($c0 \sim c35$).

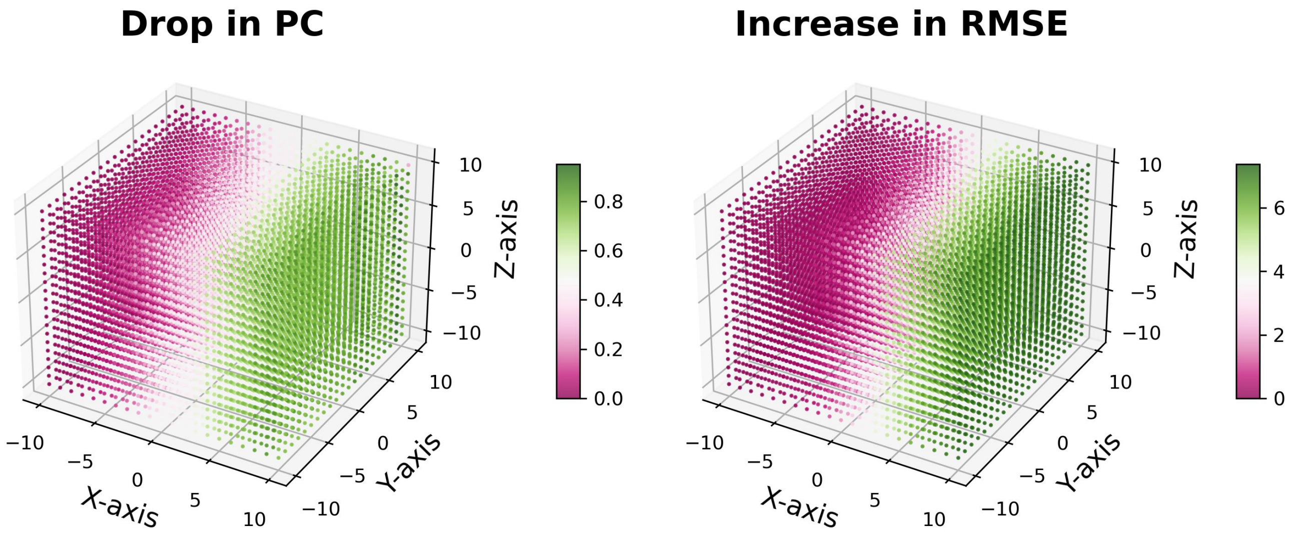

Additional file 1: Figure S2: Heatmaps showing the importance of positions, in terms of PC drop and RMSE increase, for M_{11} model. Each position is a voxel, characterized by 9 channels (hydrophobicity, hydrogen-bond donor, hydrogen-bond acceptor, aromaticity, positively ionizable, negatively ionizable, metallicity, excluded volume, and sign for a protein/ligand atom).

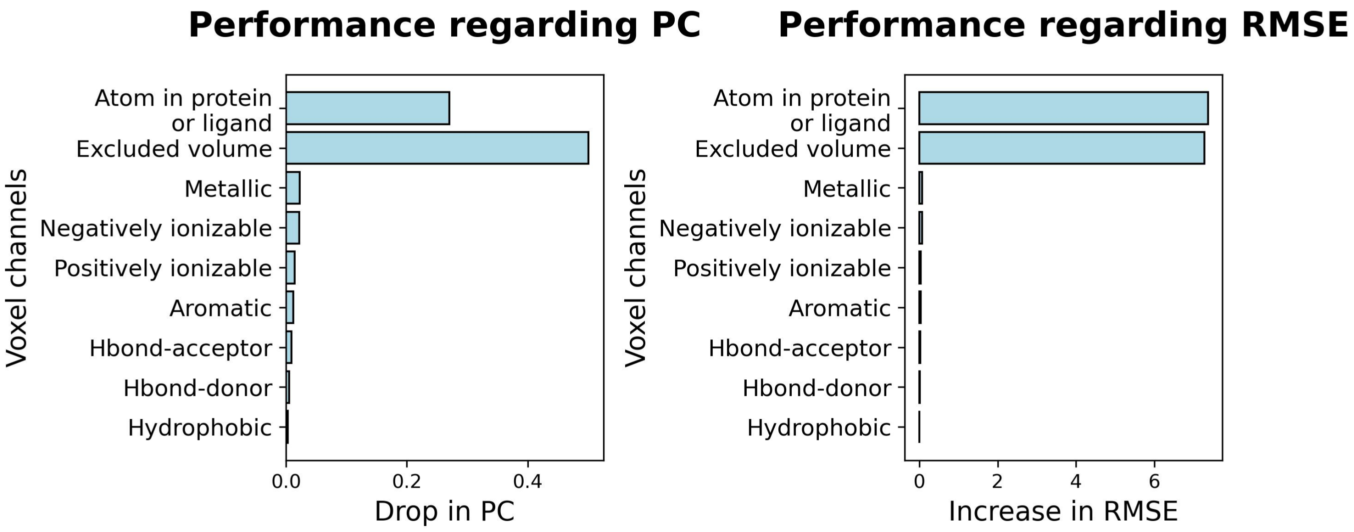

Additional file 1: Figure S3: Importance of voxel channels, in terms of PC drop and RMSE increase, for M_{11} model.

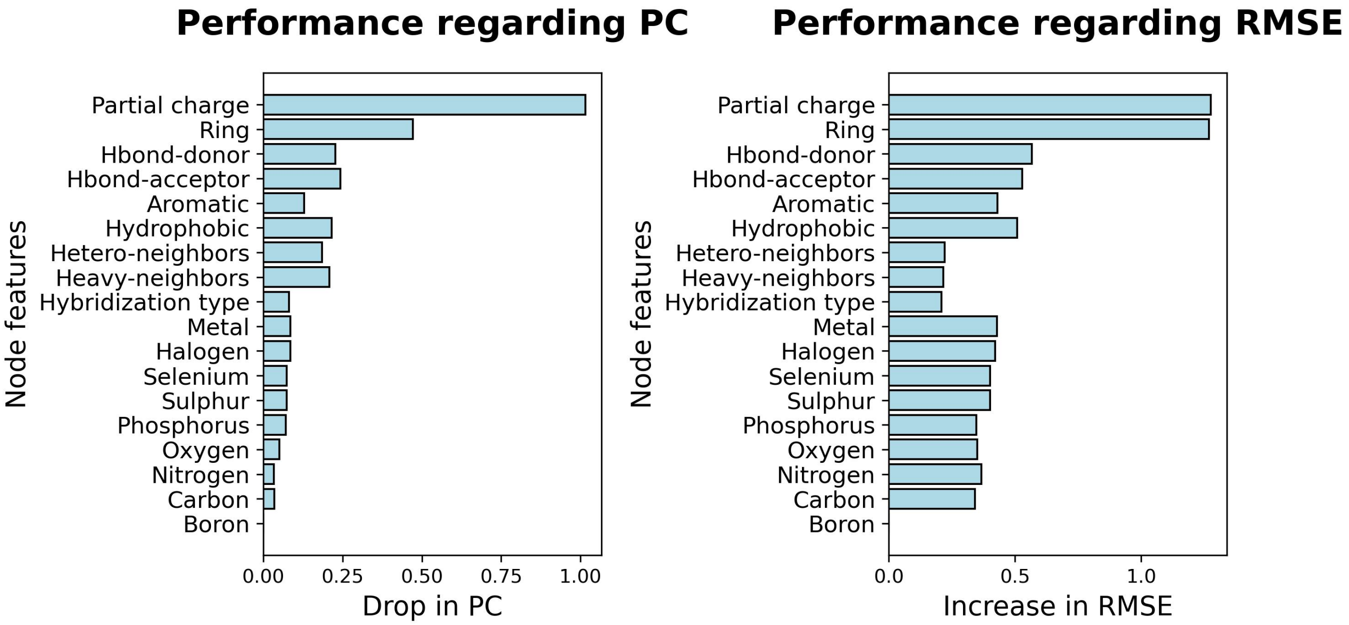

Additional file 1: Figure S4: Importance of node features, in terms of PC drop and RMSE increase, for M_{23} model.
